# Inversely Regulated Inflammation-Related Processes Mediate Anxiety–Obesity Links in Zebrafish Larvae and Adults

**DOI:** 10.3390/cells12131794

**Published:** 2023-07-06

**Authors:** Hila Yehuda, Nimrod Madrer, Doron Goldberg, Hermona Soreq, Ari Meerson

**Affiliations:** 1 MIGAL—Galilee Research Institute, Kiryat Shmona 11016, Israel; 2 The Alexander Silberman Institute of Life Sciences, The Hebrew University of Jerusalem, Jerusalem 9190401, Israel; hila.yehuda@mail.huji.ac.il (H.Y.); nimrod.madrer@mail.huji.ac.il (N.M.); 3 The Edmond and Lily Safra Center for Brain Science, The Hebrew University of Jerusalem, Jerusalem 9190401, Israel; 4 Tel-Hai College, Upper Galilee 1220800, Israel; dorongo@telhai.ac.il

**Keywords:** anxiety, antagonistic pleiotropy, caffeine, high-fat diet, immune system, obesity, noncoding RNA, RNA sequencing, transfer RNA fragments, zebrafish

## Abstract

Anxiety and metabolic impairments are often inter-related, but the underlying mechanisms are unknown. To seek RNAs involved in the anxiety disorder–metabolic disorder link, we subjected zebrafish larvae to caffeine-induced anxiety or high-fat diet (HFD)-induced obesity followed by RNA sequencing and analyses. Notably, differentially expressed (DE) transcripts in these larval models and an adult zebrafish caffeine-induced anxiety model, as well as the transcript profiles of inherently anxious versus less anxious zebrafish strains and high-fat diet-fed versus standard diet-fed adult zebrafish, revealed inversely regulated DE transcripts. In both larval anxiety and obesity models, these included long noncoding RNAs and transfer RNA fragments, with the overrepresented immune system and inflammation pathways, e.g., the “interleukin signaling pathway” and “inflammation mediated by chemokine and cytokine signaling pathway”. In adulthood, overrepresented immune system processes included “T cell activation”, “leukocyte cell-cell adhesion”, and “antigen processing and presentation”. Furthermore, unlike adult zebrafish, obesity in larvae was not accompanied by anxiety-like behavior. Together, these results may reflect an antagonistic pleiotropic phenomenon involving a re-adjusted modulation of the anxiety–metabolic links with an occurrence of the acquired immune system. Furthermore, the HFD potential to normalize anxiety-upregulated immune-related genes may reflect the high-fat diet protection of anxiety and neurodegeneration reported by others.

## 1. Introduction

Anxiety is an adaptive response to preventing future harm [1]. However, in excess and when persistent, anxiety reduces the quality of life and is considered to be a psychiatric condition, belonging to the heterogenous group termed anxiety disorders [1], which are the largest group of mental disorders in the Western world [2], with a 20% lifetime occurrence [3]. The COVID-19 pandemic has been accompanied by a greater number of anxiety cases [4], highlighting the need to explore the origins of infection-induced anxiety and seek ways to treat these devastating disorders and long COVID symptoms [5]. Intriguingly, anxiety disorders have often been found to be positively linked to obesity and metabolic syndrome. Obesity, according to the World Health Organization, is defined as abnormal or excessive fat accumulation that presents a risk to health (https://www.who.int/health-topics/obesity#tab=tab_1 (last accessed on 2 July 2023)), and metabolic syndrome is characterized by three of the following five symptoms: type II diabetes, hyperlipidemia, high LDL, low HDL, and hypertension [6]. Anxiety–metabolic syndrome/obesity links have been reported in humans [7,8], rats [9], mice [10,11,12], and adult zebrafish [13,14]. For example, in humans, a meta-analysis carried out on eighteen cross-sectional studies in four continents reflected a positive correlation between anxiety and metabolic syndrome [8]. In a longitudinal study on 2126 adult participants, baseline symptoms of anxiety were associated with a risk of abdominal obesity [7], and in a prospective study, obese children (12,507) in the Swedish Childhood Obesity Treatment Register were found to be at higher risk to suffer from anxiety disorders compared to 60,063 children in a control group [15]. Furthermore, anxiety disorders present 30–50% heritability, although specific genes or loci have yet to be found [16], and obesity is causally related to 17 human obesity loci-related genes, assessed as such in *C. elegans* [17]. The shared links between anxiety and obesity/metabolic syndrome [18,19,20] would predictably imply that genes hyperactivated in one disorder may be further elevated in the other.

Although social factors may be partially responsible for the link between obesity and anxiety, such factors cannot explain the high incidence of anxiety detected among obese persons [18]. Moreover, animal studies show definite associations between anxiety disorders and obesity/metabolic syndrome. Obese high-fat diet (HFD)-fed rats with higher fasting glucose levels than the matched controls demonstrate anxiety-like symptoms [9]. Similarly, overweight HFD-fed mice display anxiety-like behavior [10,21,22], although in one study the correlation between obesity and anxiety disappeared when attaining a certain weight [22]. Correspondingly, selectively bred obesity-prone Sprague–Dawley rats had greater anxiety-like behavior than obesity-resistant ones, but only after weight gain, regardless of their diet [23]. Additionally, HFD did not lead to anxiety-like behavior in obesity-resistant rats, even though their diet brought about weight gain and increased adiposity that was comparable to the weight gain found normally in obesity-prone rats [23]; this indicates that genetics or other factors also play a part in obesity-induced anxiety. Notably, while HFD-fed adult zebrafish showed anxiety-like behavior relative to the controls fed a regular diet [13], their five-day-old HFD-fed offspring did not exhibit anxiety-like behavior [13], hinting that obesity may correlate with anxiety-like behavior in vertebrates in an age-related manner.

Zebrafish have a few stages of development: embryos until hatching at 3–4 days post-fertilization (dpf), larvae between 4–35 dpf, juveniles between 36–90 dpf, and adults at 3 months post-fertilization, when they can reproduce [24]. These fish have become a model for many diseases since they are highly homologous to mammals, including humans, in many respects [25,26]. Genetically, 70% of the human genome has an orthologue in the genome of zebrafish, and 82% of known human morbid genes are related to at least one zebrafish orthologue [27]. Zebrafish gastrointestinal [28,29], neural [30], endocrine [28], and immune systems [31,32] have similar mechanisms of action to those of mammals. Likewise, lipid storage and insulin regulation are conserved, with obese zebrafish demonstrating a similar dysregulation of lipid metabolism to humans [28]. The anatomy, gene expression, and projections between brain regions of the teleost brain are all similar to other vertebrates [33]. Similar to humans, and unlike rodents, the zebrafish stress hormone is cortisol [30]. Its increase is associated with anxiety in both zebrafish [34] and humans [35,36]. The immune system of zebrafish has features similar to mammals, with many of the same cytokines, chemokines, and immune cell types [31]. Thus, the zebrafish is a good model to examine links between anxiety and metabolic disorders.

One manner of identifying links between anxiety and obesity would be to examine differentially expressed (DE) genes in both disorders. Such genes may be protein-coding genes, which make up less than 2% of the human genome [37], and/or genes transcribed to noncoding RNAs, including microRNAs (miRNAs) and long noncoding RNAs (lncRNAs), which can take part in the regulation of anxiety [12,38,39,40,41,42] and metabolic disorders [12,43,44,45,46,47]. Recently, a small noncoding RNA family, transfer RNA fragments (tRFs), has acquired new recognition [48,49]. As the name insinuates, these fragments are derived from enzymatic fragmentation of transfer RNAs, which occurs on pre-tRNAs and mature tRNAs. This enzymatic cleavage is not random. Under stress, the anticodon is cleaved by angiogenin in mammals or by other enzymes in other species, resulting in 5′ and 3′ tRNA halves (also termed tiRNAs) [50]. Under other circumstances (e.g., cancer), RNase Z cleaves the pre-tRNAs, producing tRF-1s, whereas dicer and other enzymes (some still unknown) divide the mature tRNAs into many types of tRFs, i.e., 5′ tRFs (tRF-5), 3′ tRFs (tRF-3), and internal tRFs (i-tRFs) [50,51,52]. tRFs, especially the 5′ halves, have been found to take part in immune system reactions [48,49]; their levels decline with age in both the cerebrospinal fluid and blood [53], and they have roles in neurological disorders [50].

The main goal of this study was to challenge the concept that specific RNA subtypes may link anxiety and obesity and identify related DE transcripts. To date and to the best of our knowledge, no single RNA-seq study has sought both coding and noncoding RNAs (ncRNA), including short regulatory RNAs, that link metabolic disorders with anxiety disorders. Our results surprisingly revealed that immune-related coding and noncoding transcripts that were upregulated in anxious vs. less anxious zebrafish were downregulated in HFD- vs. standard diet (SD)-fed zebrafish; and this inverse regulation was present in both the larval and adult stages.

## 2. Materials and Methods

### 2.1. Experimental Design

Two zebrafish larval models, one with anxiety (induced by caffeine exposure) and the other with obesity (induced by HFD) were optimized and validated, the anxiety model by behavioral and cortisol level tests and the obesity model by histology-based adiposity analysis. Subsequently, RNA was extracted, sequenced, and analyzed to identify and examine the genes that were commonly DE in both models. In addition, the effect of obesity on anxiety, and vice versa, was investigated in zebrafish larvae. We compared the larval RNA sequencing and quantitative reverse transcription–polymerase chain reaction (qRT-PCR) results with data we analyzed from adult zebrafish. The adult zebrafish data comprised the qRT-PCR results of caffeine-induced anxiety in middle-aged adult zebrafish and RNA sequencing analyses that we executed on two raw data FASTQ datasets. One dataset was of inherently anxious versus less anxious strains and the other of HFD-fed versus standard diet (SD)-fed young adult zebrafish; we compared both datasets using the same pipeline we had employed for the zebrafish larval models. Experiments with zebrafish larvae and adults were carried out under the approval of the National Council for Experiments in Animals, numbers IL-19-11-447 and IL-20-10-458, respectively.

### 2.2. Larvae and Adult Fish

Wild type zebrafish (AB strain) parents were kept under a 14 h light/10 h dark regimen with lights turned on at 8:00 am. Fish water conditions were pH 7–7.7, 27.7–28.3 °C, and 490–510 µS/cm conductivity. Parent fish were fed live 24 h-old artemia five mornings a week, and standard dry zebrafish food (Gemma micro 300, Skretting, Tooele, UT, USA) five evenings a week and one morning a week. Fish were not fed on the seventh day. Eggs were gathered and placed in Petri dishes (100 mm diameter) at a density of less than 100 per 30 mL of fish water, containing methylene blue (0.00003%). When larvae were 6 days post-fertilization (dpf), they were transferred to 2 L tanks containing 1 L of fish water (up to 100 larvae per liter of water, up to 4.5 cm deep) and fed an SD (Gemma micro 75, Skretting, Tooele, UT, USA) twice a day, five times a week, and once a day on the other two days, until 15 dpf (in behavior testing, RNA extraction or cortisol testing) or 16 dpf (in adiposity assessment).

Adult fish (15–20 months post-fertilization (mpf)) with decreased reproductive activity (i.e., produced fewer eggs) were used for the adult fish experiments. They were kept under the same conditions as the parent fish (described above) or at a density of less than 16 zebrafish in 3 L of water in tanks that were cleaned regularly. Euthanization was performed by gentle transfer and immersion in ice-cold water for at least 30 min [54,55].

### 2.3. Larval Obesity Model

Commonly, the obesity/metabolic syndrome zebrafish models that are utilized are those induced by excess nutrient, high-fat and/or high carbohydrate diets, with obesity determined by parameters such as BMI, adiposity, hyperlipidemia, and hepatic steatosis [28,56,57]. For our larval obesity model, larvae were fed an HFD based on a 20 mL hard-boiled egg yolk solution in addition to the SD. The controls were only fed the SD. The hard-boiled egg yolk solution [56] is based on yolks from chicken eggs bought fresh and boiled for 5 min. The egg yolks were mashed and added to fish water (ratio of 1 g/15 mL). After shaking the mixture vigorously for 5 min, large particles were allowed to settle out for one hour, and the egg yolk solution was divided into aliquots and frozen at −20 °C. The tanks containing the larvae were cleaned regularly and kept under the same light and temperature conditions as the parent fish. Zebrafish larvae were euthanized by immersion in ice-cold water for at least 30 min [58].

### 2.4. Caffeine-Induced Anxiety

Caffeine induces anxiety-like behavior in both zebrafish larvae [59] and adult fish [60,61]. For the larval anxiety model, we exposed larvae to a one-hour habituation period at 28 °C under light, and subsequently exposed them to caffeine (Sigma, Jerusalem, Israel). Caffeine was added as a concentrated solution to the water (final concentration 100 mg/L) containing larva(e) in wells of plates for 0.5 h at 28 °C between 9:00 a.m. and 12:30 p.m. In behavior tests, we continued caffeine exposure during the tests. In all other assays, groups of larvae, which were held in a Falcon^®^ 70 µm cell strainer (Corning, Glendale, AZ, USA) in the well of a plate, were transferred with the strainer immediately following the caffeine exposure period to ice-cold water for immobilization. Afterward, excess water was removed, and larvae were flash-frozen in liquid nitrogen. The control larvae in this model were manipulated in the same manner as the caffeine-exposed larvae but without caffeine exposure. Adult fish were exposed to caffeine (100 mg/L) for 15 min (before 1:00 pm), the acceptable duration for inducing anxiety-like behavior in adult zebrafish [60,61]. The controls were treated the same as caffeine-exposed adult fish but without caffeine exposure.

### 2.5. Larval Behavior Tests

Behavior tests were carried out to verify that caffeine induced anxiety-like behavior in larvae, as measured by thigmotaxis (the preference for the border) and erratic swimming (increased average of cumulative absolute turn angles/min). Thigmotaxis is a particularly exact measure of anxiety, as it is induced by anxiogenic drugs and ameliorated by anxiolytic treatments [59]. The larvae were distributed, one larva per well (wells of 12-well plates contained 2 mL fish water). Following habituation and caffeine exposure, the plates were transferred to the illuminated (intensity = 100%) compartment of the DanioVision instrument in the laboratory. After an additional 10 min habituation period, larval movements were videoed and recorded for 1 h. EthoVision XT 11.5 software (Noldus Information Technology, Wageningen, The Netherlands) digitalized the videos for analysis, and the parameters measured were distance moved (mm), the time duration in the center relative to the whole area (center diameter = 16.3 mm, whole area diameter = 24.1, leaving a peripheral width of 3.8 mm), and turn angle (cumulative absolute degree changes/min). The center diameter was chosen since larvae at 15 dpf are approximately 0.5 mm long [24], and the location of the larvae was determined according to their center points.

### 2.6. Adult Fish Behavior Tests

The tank (10.5 cm depth × 27 cm top length × 22 cm bottom length × 16 cm height) contained 2 L of water at a depth of 10 cm (top half of the tank = upper 5 cm layer of water). Water temperatures during the test were either 25–28 °C or in one experiment, unintentionally, 21–23 °C. Although the accepted temperature for adult zebrafish is 24–29 °C [62], their behavior at the lower temperature did not appear to differ from the higher temperature range. All behavior tests were carried out between 9:00 am and 1:00 pm. The tank was positioned inside a box (height—47 cm, width—51 cm, and depth—38 cm) that was open from one side, reducing distractions for the fish. The tank was illuminated from behind (by an A4 Graphic Tablets LED Light Box Pad Electronic USB Tracing Art Copy Board, Game Fanatics’ Store) for better resolution. Each fish was gently transferred to the tank containing water, with or without caffeine, and videoed individually for 15 min (by a digital color camera), and Debut Video Capture and Screen Recorder Software (Debut Professional v 6.63, NCH Software, Canberra, Australia) recorded the longitudinal swimming behavior (25 frames per second). The resulting videos were translated into data by a custom MATLAB script. The last 6 min of the fish behavior tests were used to evaluate anxiety-like behavior according to the parameters of height above the tank’s bottom, percent of the time in the upper half of the tank, and the number of times the fish crossed to the upper half of the tank.

### 2.7. Larval Whole-Body Cortisol Assay

Whole-body cortisol levels were determined in larvae that had or had not been exposed to caffeine (pools of 30 larvae per replicate, 15 larvae per well of 6-well plates (containing Falcon^®^ 70 µm cell strainer (Corning, Glendale, AZ, USA) and 10 mL fish water). Following caffeine exposure, the larvae were transferred with the strainer to ice-cold water, which also prevented any further increase in cortisol [63]. Cortisol was extracted according to a published procedure [63]. Briefly, 150 µL of water was added to the larval pools for homogenization by a motorized pestle and a minimum of ten subsequent passages through a syringe needle (20 G). Ethyl acetate (1 mL) was added, followed by vortex (30 s) and centrifugation (5 min, 3000× *g*, 4 °C). After freezing the aqueous layer, the solvent was transferred to a new tube and evaporated at 30 °C under nitrogen. The cortisol that remained was dissolved in a phosphate-buffered saline −0.2% bovine serum albumin (to be divided between two technical replicates) and frozen. Before continuing with an enzyme-linked immunosorbent assay (ELISA) test in a Cortisol Parameter Assay Kit (R&D Systems, Minneapolis, MN, USA), the samples were vortexed on a thermomixer for 5 min at 1100 rpm at 37 °C.

### 2.8. Adipocyte Assessment Assay

The larvae were washed and subsequently stained with Nile Red (Sigma, Jerusalem, Israel), which specifically stained neutral lipids, such as triglycerides [64]. The larvae were exposed to Nile Red (500 ng/mL) for 0.5 h and subsequently euthanized. Using a 1% low melting point agarose or a glycerol-based mounting medium (ibidi Mounting Medium, ibidi, Grafelfing, Germany), each larva was positioned on its right side (location of adipocyte deposit) on a coverslip assembly, based on a published diagram [65] (wiki.zfin.org/display/prot/Viewing+Chambers, last accessed on 2 July 2023). The degree of obesity was assessed by the number of Nile Red-stained lipid droplets (assumed to be in adipocytes) and their area, as determined by confocal microscopy (Zeiss LSM 700 laser scanning confocal microscope, using an X5 objective, laser settings of 488 nm for excitation and ≥539 nm for emission and ZEN 2.6 (blue edition) software (Zeiss)). Conditions, including the laser power, pinhole, master gain, and digital offset were kept constant for each experiment. The layer counted was the one with the most Nile Red-stained lipid droplets in the abdominal area. Lipid droplets smaller than 100 µm^2^ were excluded, as were those appearing in a row (since they could be fat droplets in lymph vessels and not adipocytes).

### 2.9. Caffeine Pretreatments

In the experiments examining the effect of anxiety on obesity, the larvae were pretreated with caffeine and assayed for the accumulation of adipocytes on day 15 post-fertilization (pf), as described above. The time of day, concentration, and duration of exposure to caffeine were the same as the other assays, but treatments were performed either on day 7, days 5 and 9, or days 5, 7, 9, and 12 pf, in the one, two, and four caffeine pretreatment experiments, respectively. For the pretreatments, larvae (up to 25) were gently concentrated in 50 mL of water, which was added or not added to a concentrated solution of caffeine. Following the half-hour exposure to caffeine (0 or 100 mg/L), the larvae were gently transferred to their original tank using a 150 µm filter.

### 2.10. RNA Extraction

RNA was isolated from (1) pools of larvae and (2) whole bodies or individual organs of adult fish. All extractions were preceded by homogenization using a motorized pestle and at least 10 subsequent passages through a syringe needle (20 G). Extraction from adult whole bodies (minced) or a small portion of the tail area (skin, muscle, and bone) was performed by QIAzol Lysis Reagent according to the Qiazol Protocol (Qiagen, Hilden, Germany), from livers and intestines of the adult fish using a Tissue Total RNA Mini Kit (Geneaid Biotech Ltd., Taiwan), and from the larvae and brains using Nucleospin^®^ miRNA kit (Macherey-Nagel GmbH & Co. KG, Duren, Germany). All extractions were according to the manufacturer’s recommendations.

### 2.11. RNA-Seq

Twelve RNA samples were sequenced. Each larval sample was a biological replicate derived from a pool of 30 larvae (15 dpf). There were three replicates per treatment and four treatments: no treatment (NO) and caffeine (CAF) in the anxiety model and standard diet (SD) and high-fat diet (HFD) in the obesity model. The CAF samples were larvae that had been exposed to caffeine in groups of fifteen larvae per well of 6-well plates (containing Falcon^®^ 70 µm cell strainer (Corning, USA) and 10 mL fish water) and two wells per sample. The NO samples were the controls of the CAF samples and, therefore, were manipulated in the same manner, but with no treatment. The SD samples were the controls of the HFD-fed larvae. The larvae of these samples were fed either SD or HFD from 6 dpf until 15 dpf. Subsequently, they were collected, immobilized, and flash-frozen in the same manner as the NO and CAF larvae. The samples were kept at −80 °C until RNA extraction.

Integrity assessment with an Agilent TapeStation, library preparation, and sequencing of the RNA was carried out at the Technion Genomics Center, Technion-Israel Institute of Technology. The samples had an RNA integrity number (RIN) > 8.5, except for the HFD samples, with RINs of 7.8–8.2, possibly because of the high-fat content. Twelve Poly(A)+ and twelve short RNA libraries were prepared. The short RNA libraries were generated using a QIAseq miRNA Library Kit, and the Poly(A)+ RNA libraries were generated using a TruSeq RNA Library Prep Kit v2, both according to the manufacturer’s protocol. Sequencing was performed on Illumina HiSeq 2500, generating 75 nucleotide single-end runs from the short RNA libraries and 51 nucleotide single-end runs from the poly(A)+ RNA libraries.

The poly(A)+ RNA libraries yielded from 14,745,028 to 20,885,465 reads per sample. As assessed using FastQC, version v0.11.9, the per base sequence quality was >32, except for base 5, in all sequences. None of the sequences had overrepresented sequences or adapter content. Trimming (Trimmomatic v. 0.36) was not carried out, since it did not improve sequence quality, according to FastQC. The zebrafish reference genome used for mapping was Danio_rerio.GRCz11.dna.primary_assembly.fa.gz (the primary assembly of the genome, which contains all top-level genes. Haplotypes were excluded to simplify analysis (https://ftp.ensembl.org/pub/release-98/fasta/danio_rerio/dna/README, last accessed on 2 July 2023). For annotation, Danio_rerio.GRCz11.98.gtf.gz was used. We used RSEM software v. 1.3.1 for read alignment (BOWTIE2), annotation, and expression calculation (https://bioinformaticshome.com/tools/rna-seq/descriptions/RSEM.html, last accessed on 2 July 2023). The RSEM model statistics, obtained by RSEM for the 12 samples, showed that the fragment lengths were similar (51 ± 0.7, mean ± sd), the read lengths were identical (51 ± 0), the observed quality vs. Phred Quality score graphs were similar, and the alignment statistics were also similar (48–50% multialigned, 34–36% unique, 15–18% unalignable).

A DE analysis of RNA isoforms was carried out by the RSEM wrapper of EBSeq. An ngvector was generated to group information, which is necessary for EBSeq isoform-level differential expression. The resulting EBSeq output was a matrix with normalized mean counts for each transcript of each treatment. Those genes that had a false discovery rate (FDR) < 0.05 were obtained by the RSEM wrapper of FDR. In EBSeq, there are 15 patterns from which one can determine DE patterns, which are relevant for determining isoforms that were DE in both the anxiety model and the obesity model. Pattern 4—Caf and HFD are the same but different from SD and NO, which are the same; pattern 9—Caf and HFD are the same but different from SD and NO, which are different; pattern 14—Caf and HFD differ, but SD and NO are the same; and pattern 15—all treatments differ. These pattern subsets were determined, and fold changes were calculated. Isoforms were considered to be DE if the fold change had an absolute log2 > 0.58 (in real numbers—>1.5 or <0.67) and an FDR < 0.05.

In addition, mRNA transcriptomic analysis was carried out using DESeq2 (https://bioconductor.org/packages/release/bioc/html/DESeq2.html (last accessed on 2 July 2023)) in Bioconductor 3.10 (https://www.bioconductor.org/install/ (last accessed on 2 July 2023)) and R software 3.6.1 (https://www.r-project.org/ (last accessed on 2 July 2023)). Genes were considered to be DE if they had a significance of *p* < 0.05 and a fold change > 1.5 fold or <0.67. Gene functions were analyzed by the overrepresentation test of Panther Gene Ontology, version 17.0 (http://pantherdb.org/geneListAnalysis.do (last accessed on 2 July 2023)); those used were Panther GO-slim biological process (BP), Panther GO-slim molecular functions (MF), Panther GO-slim cellular components (CC), Panther Pathways, Reactome Pathways, and Panther Protein Class. In these tests, genes are considered to be overrepresented if they appear significantly more than in the Danio rerio reference genome (and they are considered to be underrepresented if they appear significantly less). The following gene groups were tested as pairs to find the GO-terms and pathways that were commonly overrepresented in both: upregulated (FC > 0, padj < 0.05) anxiety and obesity DE gene lists; upregulated anxiety DE genes with downregulated (FC < 0, padj < 0.05) obesity DE genes; and downregulated anxiety DE genes with each of these two obesity DE gene lists. The RNA-seq poly(A)+ results have been deposited at the database repository Gene Expression Omnibus (GEO), as SuperSeries GSE207550/SubSeries GSE207457.

Short RNA quality was analyzed by the Technion Genomics Center. FastQC version 0.11.5 demonstrated that the reads had a high quality, i.e., Phred Score > 33 and 26,126,096 to 38,085,377 reads per sample.

For miRNA analysis, the reads were trimmed using the online CLC Genomics Workbench 20.0.3, which included the removal of the Qiagen 3′ adapter on the 3′ end. Reads without this adapter were discarded, and only sequences between 15 and 54 nucleotides were retained. These trimmed files (read lengths of ~23 bases) were joined. miR quantify was run on these files using the reference miRbase v.22.1 and the preference Danio rerio. Normalization was executed using TMM (trimmed mean of M values; TMM normalization adjusts library sizes based on the assumption that most genes are not DE). Annotation was carried out using Go Mapping RNAcentral v10. The miRNA-seq results have been deposited at GEO, as SuperSeries GSE207550/SubSeries GSE207549.

For the tRF analysis, the short RNA sequencing was aligned to tRFs using MINT-map [66], according to the default settings of the pipeline. All tRFs for which the 75th quantile was lower than the mean over all samples were removed from the count data a priori (13 tRFs were excluded). Differential expressions of tRFs were assessed using the edgeR package [67].

### 2.12. cDNA Synthesis

For mRNA and lncRNA, the Verso cDNA Synthesis Kit (ThermoFisher Scientific, Waltham, MA, USA) was employed to synthesize cDNA for qRT-PCR from the RNA extracted above. It was carried out based on the manufacturer’s recommendations. Briefly, RNA (0.25–1 µg) was heated to 70 °C for 5 min and cooled to 4 °C before adding the anchored-oligo dT primer and other reagents supplied in the kit, including the RT enhancer that prevents genomic DNA carryover. The cDNA synthesis reaction was executed at 42 °C for 60 min, followed by 52 °C for 30 min, and lastly, enzyme inactivation at 95 °C for 2 min.

For miRNAs, cDNA was synthesized based on a published protocol [68]. Briefly, the 10 µL reaction, which included 1 µg RNA, 1 µL of 10X poly(A) polymerase buffer (New England Biolabs, Ipswich, MA, USA), 0.25 mM ATP, 1 mM dNTPs (New England Biolabs, USA), 1 µM RT-primer (GGTCCAGTTTT TTTTTTTTTTTVN (V is A, C, and G; N is A, C, G, and T)), 80 U MuLV reverse transcriptase (New England Biolabs, USA), and 0.75 U poly(A) polymerase (New England Biolabs, Ipswich, MA, USA) were subjected to 42 °C for 1 h, followed by enzyme inactivation at 95 °C for 5 min.

### 2.13. qRT-PCR

cDNA was amplified in a reaction that contained the iTAQ Universal SYBR Green Supermix (Bio-Rad Laboratories, Haifa, Israel) (based on the protocol supplied by the manufacturers) and specific primers (Merck, Rehovot, Israel) (Table S1) in the 7900HT Applied Biosystems instrument. Primers for mRNA and lncRNA were designed using the NCBI Primer-Blast site (last accessed on 2 July 2023), and primers for miRs were designed according to Busk [69]. For normalization in mRNA and lncRNA qRT-PCR, the average of the reference genes, Danio rerio eukaryotic translation elongation factor 1 alpha 1, and like 1 (eef1a1l1, NM_131263.1), as well as Danio rerio actin and beta 2 (actb2, NM_181601.5), were used. These genes have been reported to be good references for qRT-PCR in zebrafish [70]. For miR qRT-PCR, the average of the three miRs, dre-miR-let7a, dre-miR-125b-5p, and dre-miR-26a-5p, was employed for normalization. These miRs were chosen since their absolute fold change was ≤1.1 according to the small RNA-seq analysis using CLC Genomics Workbench v.20. The PCR cycles were as follows: denaturation at 95 °C for 1 min followed by 40 cycles of 95 °C for 15 s and 58 °C for 30 s. An analysis was performed according to the 2^−ΔΔCt^ method.

### 2.14. Datasets

#### 2.14.1. Brains of Anxious vs. Less Anxious Adult Zebrafish

We further carried out a transcriptomic analysis of brain samples from a publicly available dataset (accession number GSE61108) of adult zebrafish with different inherent levels of anxiety-like behavior. The strains in the dataset included AB (also used in the above experiments), which is a low anxiety-like strain, common in laboratories, and two other strains with higher anxiety-like behavior, termed here collectively as SB, comprising LSB (low stationary behavior) and HSB (high stationary behavior) [71,72]. Each strain was represented by four pooled samples of ten whole brains (two males and two females); thus, for our analysis, there were four AB samples and eight SB samples (four HSB and four LSB). cDNA libraries had been generated with TruSeq RNA Sample Prep V2 of Illumina and sequenced as 72 bp single ends on Illumina (GA_IIX_), similar to our procedures. To analyze this dataset, we used a similar procedure to the RNA analysis for larval samples, with one exception. FastQC, version v0.11.9 revealed TruSeq adapters, which we removed with Trimmomatic v.0.36 and further confirmed removal by FastQC analysis. The remaining analysis was executed with RSEM and DESeq2, as described above.

#### 2.14.2. Telencephalons of HFD-Fed vs. SD-Fed Adult Zebrafish

A dataset of transcripts from 3 months post-fertilization of male zebrafish telencephalons, as described in the article [73], was kindly provided by Dr. Shinichi Meguro from the Kao Corporation, Tokyo, Japan. In brief, the fish were fed an HFD of 80% control diet and 20% lard (*w*/*w*) or the SD (control diet) for 11 weeks. Euthanization was by immersion in tricaine for the dissection of telencephalon. The libraries had been prepared using the TruSeq RNA Sample Preparation Kit v2 of Illumina and sequenced on the HiSeq 2500 platform (Illumina Inc., San Diego, CA, USA) as 101 bp paired ends. We used FastQC version v0.11.9 to analyze read quality and Trimmomatic v.0.36 to remove any adapters. Their removal was confirmed by FastQC analysis. The remaining analysis was executed with RSEM and DESeq2, as described above.

### 2.15. Statistical Analysis

Statistical analyses for larval behavior tests and whole-body cortisol levels were conducted using the Welch Two Sample *t*-test and the Wilcoxon Test, and adiposity analyses were carried out by the Welch Two Sample *t*-test, all by R software 3.6.1 (https://www.r-project.org/ (last accessed on 2 July 2023)). Adult zebrafish behavior tests and qRT-PCR transcript and miRNA levels were statistically analyzed using the two-tailed Student’s *t*-test. Gene DE (not isoforms or short RNAs) was analyzed by DESeq2 in R, whereas DE analysis of the long RNA isoforms was carried out by the RSEM wrapper of EBSeq. The RNA isoforms were determined to be DE according to the RSEM wrapper of FDR. CLC Genomics Workbench 20.0.3 was used for miRNA statistical analyses, and EdgeR [67] was employed for the statistical analysis of the tRFs. Boxplots were plotted using ggplot2 [74]. Gene functions that were analyzed according to the overrepresentation test of Panther Gene Ontology, version 17.0 (http://pantherdb.org/geneListAnalysis.do (last accessed on 2 July 2023)) were considered to be significantly overrepresented or underrepresented, according to Fisher’s exact test, with FDR correction.

## 3. Results

### 3.1. Experimental Validation of the Anxiety and Obesity Models

To investigate transcriptomic profile modifications induced in anxiety and obesity, 15 dpf zebrafish larvae were used in two well-known models. The anxiety model was based on a short exposure (0.5 h) to a high caffeine dose (100 mg/L) [59,60,61], and the obesity model was induced by an egg yolk-based high-fat diet [28]. Caffeine-exposed larvae (CAF) compared to non-treated larvae (NO) showed greater anxiety-like behavior, including increased thigmotaxis (duration in the center—means ± SEM were 7.4 ± 2.4% for CAF vs. 24.9 ± 4.3% for NO, Welch Two Sample *t*-test, *p* = 0.002) and erratic swimming (cumulative average absolute turn angle (degrees/min) were 31.2 ± 1.7 for CAF vs. 21.2 + 0.8 for NO, Welch Two Sample *t*-test, *p* = 0.00008) (Supplementary Figure S1A,B,E), as well as higher cortisol levels (means ± SEM were CAF—120 ± 13, NO—55 ± 6 pg/larvae, Welch Two Sample *t*-test, *p* = 0.002) (Figure S1F). High cortisol levels are known to correlate with anxiety in zebrafish [34]. The caffeine-exposed larvae also had reduced locomotion (Figure S1C), which was in agreement with published studies [60,61]. In the obesity model, two independent experiments (*n* = 24–25) revealed a greater percentage of adipocyte-laden larvae among high-fat diet (HFD)-fed larvae compared to standard diet (SD)-fed larvae (56% vs. 21%, respectively). In addition, HFD-fed larvae had more abdominal adipocytes than SD-fed larvae (means ± SE—2.12 ± 0.50 vs. 0.50 ± 0.23 adipocytes/larva, respectively; Welch Two Sample *t*-test, *p* = 0.006) (Figure S1G), and the average total area of adipocytes in HFD-fed larvae was larger than SD-fed larvae (means ± SE—1973 ± 447 µm^2^ versus 222 ± 118 µm^2^, respectively; Welch Two Sample *t*-test, *p* = 0.0008) (Figure S1H). Together, these results validate the efficacy of the anxiety and obesity models.

### 3.2. Most Transcripts That Were Upregulated in Anxious Larvae Were Downregulated in Obese Larvae

The concept of RNAs linking anxiety and obesity and identifying DE genes was explored by subjecting RNA from larvae of the two models to next-generation RNA sequencing. The RNA sequence of each model comprised the control and treatment sets. NO versus CAF for the anxiety model and SD versus HFD for the obesity model, with 30 larvae per replicate and three replicates per treatment.

**Figure 1:**
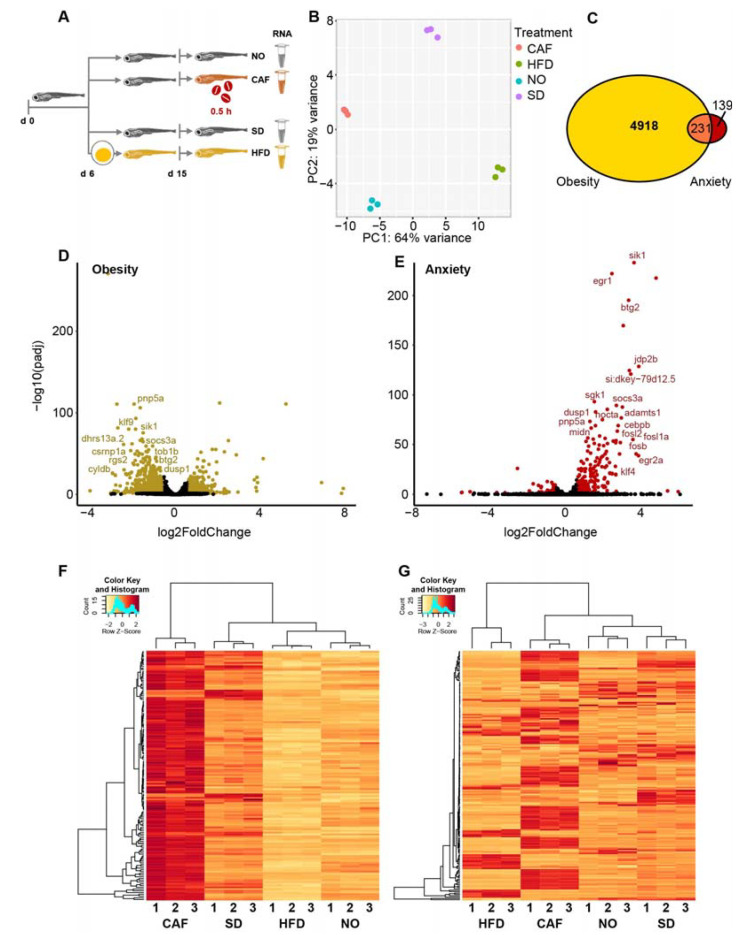
Most anxiety-upregulated larvae transcripts were downregulated in obesity. (**A**) Experimental design: zebrafish larvae (6 days post-fertilization (dpf)) were fed either a standard diet (SD) or high-fat diet (HFD, based on hard-boiled egg yolk solution). At age 15 dpf, a group of the SD-fed larvae were further divided into caffeine-exposed larvae (CAF) and the controls (non-treated (NO)). Following caffeine exposure (0.5 h) of the CAF larvae, larvae of all treatments were snap-frozen until RNA extraction for RNA-seq. (**B**) Profiles based on 32,520 Poly(A)+ mapped transcripts clustered according to principal component analysis (PCA) treatment. (**C**) A total of 231 poly(A)+ transcripts were differentially expressed (DE) (padj < 0.05) in both the obesity (yellow) and anxiety (red) models. (**D**) Obesity model volcano plot—DE, transcripts color-marked gold; padj < 0.05 and absolute log2 fold change > 0.58 (>1.5 and <0.67-fold change)). Labeled transcripts are among the 107 genes upregulated > 1.5 fold in the anxiety model and downregulated < 0.67 fold in the obesity model. (**E**) Anxiety model volcano plot—DE, transcripts color-marked red; padj < 0.05 and absolute log2 fold change > 0.58 (>1.5 and <0.67-fold change)). Labeled transcripts are among the 107 genes upregulated > 1.5 fold in the anxiety model and downregulated < 0.67 fold in the obesity model. (**F**) Heatmap of transcripts per million (TPM) from RSEM of 142 DE poly(A)+ transcripts upregulated in anxiety model (>1.5 fold, padj < 0.05). (**G**) Heatmap of 182 DE isoform transcripts (FDR < 0.05) that were upregulated > 1.5-fold in the anxiety model and downregulated < 0.67-fold in the obesity model. A total of thirty larvae per replicate, three replicates per treatment.

Following RSEM annotation and expression calculation, DESeq2 was used for normalization and statistics. According to DESeq2, among the 32,520 poly(A)+ genes, including all mRNA and many lncRNA genes, there were 29,720 genes with a non-zero read count. Principal component analysis (PCA) showed that the levels of all (32,520) poly(A)+ transcripts clustered according to treatment (Figure 1A,B). Thus, the biological replicates had similar expression profiles, and these profiles differed among treatments. The two controls, SD and NO, probably varied due to larvae being transferred to wells containing filters for quick removal following caffeine exposure, which was not carried out on the SD (and HFD) larvae.

There were 5149 (17.3%) DE genes in the obesity model compared to merely 370 (1.2%) in the anxiety model (padj < 0.05) (Figure 1C). The difference may indicate a greater effect of HFD-induced obesity versus caffeine-induced anxiety on gene expression. Furthermore, DE genes in the obesity model were mostly downregulated, whereas DE genes in the anxiety model were mostly upregulated (Figure 1D,E). This inverse regulation was even more prominent when analyzing the 231 genes commonly DE (padj < 0.05) in both models (Figure 1C), i.e., their direction of change was almost totally inverse; 142 genes were upregulated in the anxiety model, but only 10 genes were upregulated in the obesity model (fold change > 1.5). Inversely, 143 genes were downregulated in the obesity model, with only 23 genes being downregulated in the anxiety model (fold change < 0.67). Thus, among the 142 genes that were upregulated in the anxiety model, 107 (75%) were downregulated in the obesity model. These DESeq2-analyzed results are reflected in the heatmap (Figure 1F). Of the 231 intersecting genes, only 1 gene was upregulated in both models (*foxq1a*, fold change > 1.5), and 8 genes were downregulated in both (*si:ch211-125o16.4*, *pglyrp6*, *osgn1*, *apoda.2*, *ins*, *urgcp*, *arid6*, and *CABZ01046997.1*; fold change < 0.67). We summarize that fish larvae show inverse profiles of DE genes in the obesity and anxiety states.

### 3.3. DE Isoform Results Were Similar to DE Gene Results

DESeq2 analyzes the expression of genes, but not isoforms. If one isoform of a gene is upregulated and another isoform of the same gene is downregulated to the same degree, the DE information is lost since the two changes cancel each other out. According to EBSeq analysis, there were 52,061 mapped isoform transcripts, including mRNAs (41,203), retained intron long noncoding (lncRNAs (3107)), processed transcript lncRNAs (2841), long intergenic noncoding RNAs (lincRNAs (1901)), antisense lncRNAs (754), sense intronic lncRNAs (54), sense-overlapping (9), and other ncRNAs (Table S2), and of these mapped isoform transcripts, 10,688 were DE (FDR < 0.05). Among the DE transcripts, 473 were DE isoform transcripts that belonged to the selected DE patterns, appropriate for DE in the anxiety and obesity models (Section 2). Of these, 182 had a fold change of either > 1.5 or <0.67 in both models, clustering according to treatment (Figure 1G). Similar to the gene transcriptomics analysis above, 92 of the 182 isoforms (51%) that were upregulated in the caffeine-exposed larvae were downregulated in the HFD-fed larvae. Thus, isoform transcripts, as well, showed inverse regulation patterns in anxious and obese fish larvae.

### 3.4. Inflammation/Immune System Protein-Coding Transcripts Are Upregulated in Anxious Larvae and Downregulated in Obese Larvae

**Figure 2:**
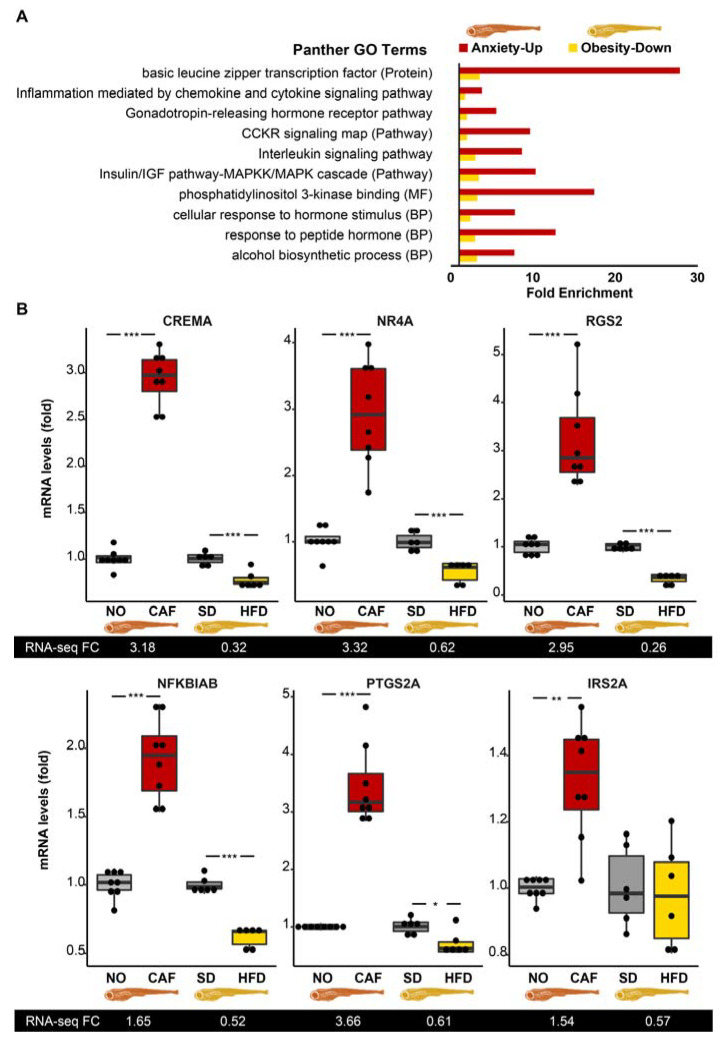
Larval inflammation/immune pathways are upregulated in anxiety and downregulated in obesity. (**A**) PANTHER Overrepresentation Test identified “CCKR—gastrin- and cholecystokinin-mediated regulation of cellular processes signaling map”, “inflammation mediated by chemokine and cytokine signaling pathway”, and “interleukin signaling pathway”. Reference genes were all those included in the Danio rerio database, Fisher’s exact test, correction by FDR < 0.05. (**B**) Quantitative reverse transcription polymerase chain reaction (qRT-PCR) results of the anxiety and obesity models are presented as graphs, with RNA-seq results below each graph, *n*= 6–8 replicates (10–30 larvae/replicate), 2–3 separate experiments, Student’s *t*-test. Reference genes are the average of eef1a1l1 and actb2. *—*p* < 0.05, **—*p* < 0.01, ***—*p* < 0.001.

Next, we explored the overrepresented gene ontology (GO) terms (Fisher’s Exact Test, FDR < 0.05) of the anxiety-upregulated (238 mapped/281) and downregulated [73/89] transcripts, and the obesity-upregulated (1993/2396) and downregulated (2331/2753) transcripts, padj < 0.05) ones. Most notable among the anxiety-upregulated and obesity-downregulated terms were the immune-related protein class, “basic leucine zipper transcription factor” (involved in the development of immune cells [75]), and the three immune system-related pathways: “inflammation mediated by chemokine and cytokine signaling pathway”, “CCKR (gastrin- and cholecystokinin-mediated regulation of cellular processes) signaling map”, and the “interleukin signaling pathway” (Figure 2A). The latter two were also overrepresented when the fold change threshold of the genes included in the lists was changed to >1.5 fold and <0.67 fold. In comparison, digestion-associated Reactome Pathways, i.e., the “metabolism of water-soluble vitamins and cofactors”, the “degradation of the extracellular matrix”, and “digestion” were common among the two downregulated gene lists (Figure S2A). Comparing the two upregulated lists revealed that the highest fold change GO-slim biological process (BP) terms comprised the “small molecule biosynthetic process” and the “response to organonitrogen compound” (Figure S2B). The only commonly overrepresented term in the anxiety-downregulated and obesity-upregulated comparison was the GO-slim BP “macromolecule metabolic process” (Figure S2C). More results derived from the comparison of the anxiety-upregulated and obesity-downregulated genes included the following GO-slim BP terms: “response to peptide hormone”, “cellular response to hormone stimulus”, and “alcohol biosynthetic process”. We conclude that the dominant change in both experimental models involved changes in immune/inflammatory regulation, which were exacerbated under anxiety and suppressed in obesity.

The immune/inflammatory-related pathways noted above included a total of 99 genes, with 14 (13%) being among the inversely regulated 107 anxiety–obesity genes, i.e., early growth response 1 (*egr1*, also a cholinergic transcription factor in innate immune-related cells [76]), nuclear receptor subfamily 4 group A member 1 (*nr4a1* aka *NUR77*), serpin-family E member 1 (*serpine1*), cAMP responsive element modulator a (*crema*), regulator of G protein signaling (*rgs*)*13*, prostaglandin–endoperoxide synthase 2a (*ptgs2a* aka *COX2a*), *rgs2*, NFκB inhibitor α b (*nfkbiab*), insulin receptor substrate *(irs)2a, irs2b*, chemokine (C-C motif) receptor 9a (*ccr9a*), MYC proto-oncogene (*mycb*), interleukin 4 receptor, tandem duplicate 1 (*il4r.1*), and chemokine (C-X-C motif) receptor 4b (*cxcr4b*), in at least one of the pathways. Six of the fourteen inflammation/immune-related DE transcripts were tested and successfully validated by qRT-PCR (Figure 2B). Most of these transcripts may suppress and/or induce inflammation, depending on the conditions [77,78,79,80,81,82,83]. This further suggested that the seemingly inverse roles of these regulators may hence be subject to additional control (e.g., via noncoding RNAs) under stress conditions.

The remaining 93 genes were enriched for biological processes related to ectoderm, mesoderm and endoderm formation (83-fold), negative regulation of MAPK cascade (27-fold), positive regulation of proteolysis (18-fold), regulation of protein serine/threonine kinase activity (16-fold), MAPK cascade (8-fold), regulation of cell cycle (5-fold), regulation of transcription by RNA polymerase II (3-fold) (Figure S3A), and the p53 Pathway (22-fold) (Figure S3B).

### 3.5. lincRNAs Upregulated in the Anxiety Model Were Downregulated in the Obesity Model

**Figure 3:**
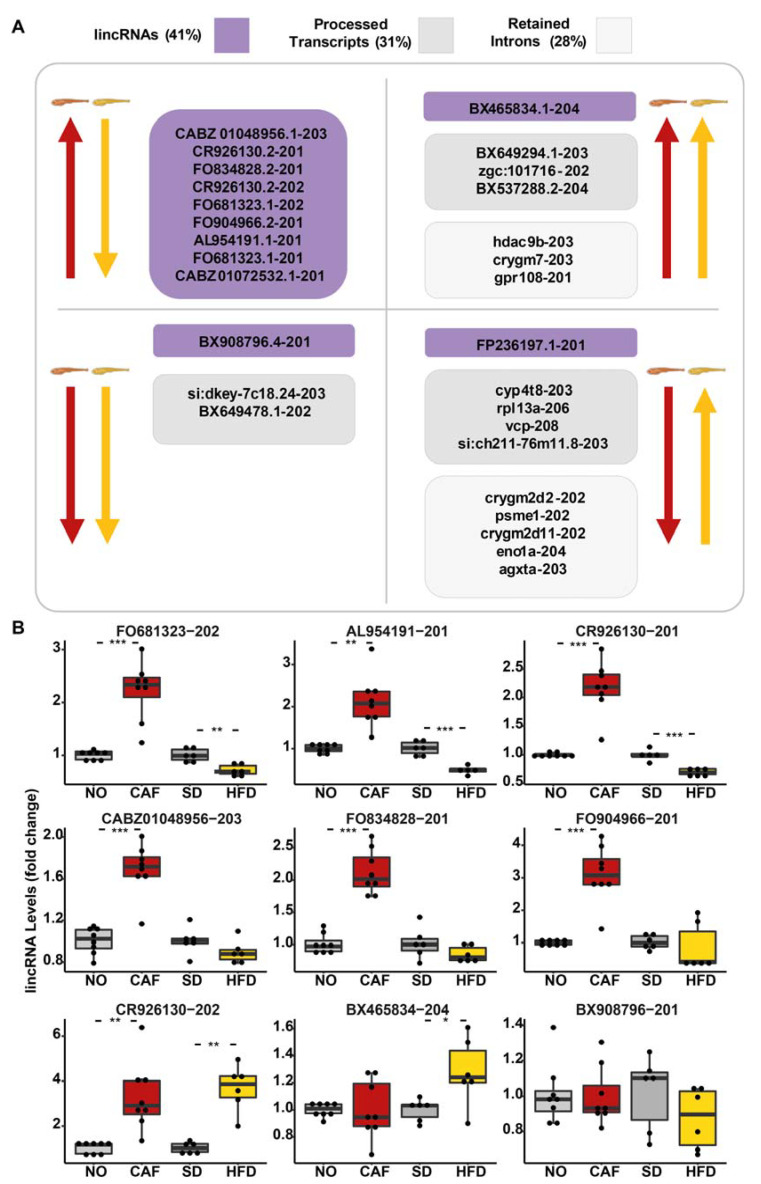
Most differentially expressed (DE) long intergenic noncoding RNAs (lincRNAs) were upregulated in the anxiety model and downregulated in the obesity model, similar to protein-coding genes. (**A**) Long noncoding RNAs (lncRNAs), classified as long intergenic noncoding RNAs (lincRNA) (purple), processed transcripts (dark grey), and retained introns (light grey) with fold change > 1.5 or <0.66, FDR < 0.05. Arrows pointing upward: upregulation, arrows pointing downward: downregulation. Yellow: obesity model; red: anxiety model. (**B**) qRT-PCR of lincRNA. *N* = 5–8 replicates (10–30 larvae/replicate), 2–3 separate experiments. Reference genes: average of eef1a1l1 and actb2. *—*p* < 0.05, **—*p* < 0.01, ***—*p* < 0.001. Student’s *t*-test.

In addition to coding sequences, the 182 DE isoform transcripts in both models (FDR < 0.05, fold change > 1.5 and <0.67) included twenty-nine noncoding poly(A)+ RNAs, that could be divided into three classes (according to Biomart Ensembl): twelve long intergenic noncoding RNAs (lincRNAs) (41%), nine processed transcripts (31%), and eight retained introns (28%) (Figure 3A). Similar to the pattern found for DE poly(A)+ mRNAs, most of the lincRNAs that were upregulated in the anxiety model were downregulated in the obesity model. The DE lincRNAs, identified by RNA-seq, were tested by qRT-PCR (Figure 3B), validating *FO681323.1-202*, *AL954191.1-201*, and *CR926130.2-201* upregulation in the anxiety model and downregulation in the obesity model. *CABZ01048956-203*, *FO834828-201*, and *FO904966-201* also tended to be inversely regulated. As these molecules exhibited a regulation pattern similar to most DE protein-coding genes in this study, and since lincRNAs are known to have regulatory activities [84], we may speculate that these molecules also take part in the regulation of inflammatory pathways. To our knowledge, this is the first report on the possible functions of these transcripts.

### 3.6. The Processed Transcript, si:dkey-7c18.24-203, Was Downregulated in Both the Anxiety and the Obesity Models According to RNA-Seq and qRT-PCR

Of the retained intron and processed transcript lncRNAs that were DE according to RNA-seq, only those upregulated in both models or downregulated in both models were tested by qRT-PCR. None of the RNA-seq results of the retained intron lncRNAs (Figure S4A,B) were confirmed by qRT-PCR. However, one of the processed transcripts (Figure S4C–F), *si:dkey-7c18.24-203*, was downregulated by about 30% in both models according to qRT-PCR (Figure S4F), validating its RNA-seq results.

### 3.7. Neighboring mRNAs of DE lincRNA Did Not Imply Functions for lincRNA

Notably, lncRNAs may regulate neighboring mRNAs transcribed from genomic regions within 100 kb of them [85], which could imply a possible function. The chromosome number, strand, transcript length, exon number, and gene length of the DE lincRNAs are presented in Table S3. To locate the mRNAs that were within 100 kb of the lincRNAs, we used Genome Browser online software (GRCz11/danRer11) (https://genome.ucsc.edu/ (last accessed on 2 July 2023)). To find predicted orthologues with high scores of these mRNAs, we utilized the DRSC Integrative Ortholog Prediction Tool v. 8 (DIOPT) (https://www.flyrnai.org/cgi-bin/DRSC_orthologs.pl (last accessed on 2 July 2023)) (Table S4). The human mRNA orthologues found included *calmegin*, *NAA15* (also called NMDA receptor-regulated gene 1b), and *H2AZ2* (or h2afv, H2A histone family-member V). However, of these genes, only *H2AZ2* was DE (padj < 0.05, FC > 1.5 or <0.67) and was only in the obesity model. Therefore, neighboring mRNAs were not informative as to the possible functions of the lincRNAs.

### 3.8. miRNAs Do Not Seem to Link Anxiety and Obesity in Zebrafish Larvae

**Figure 4:**
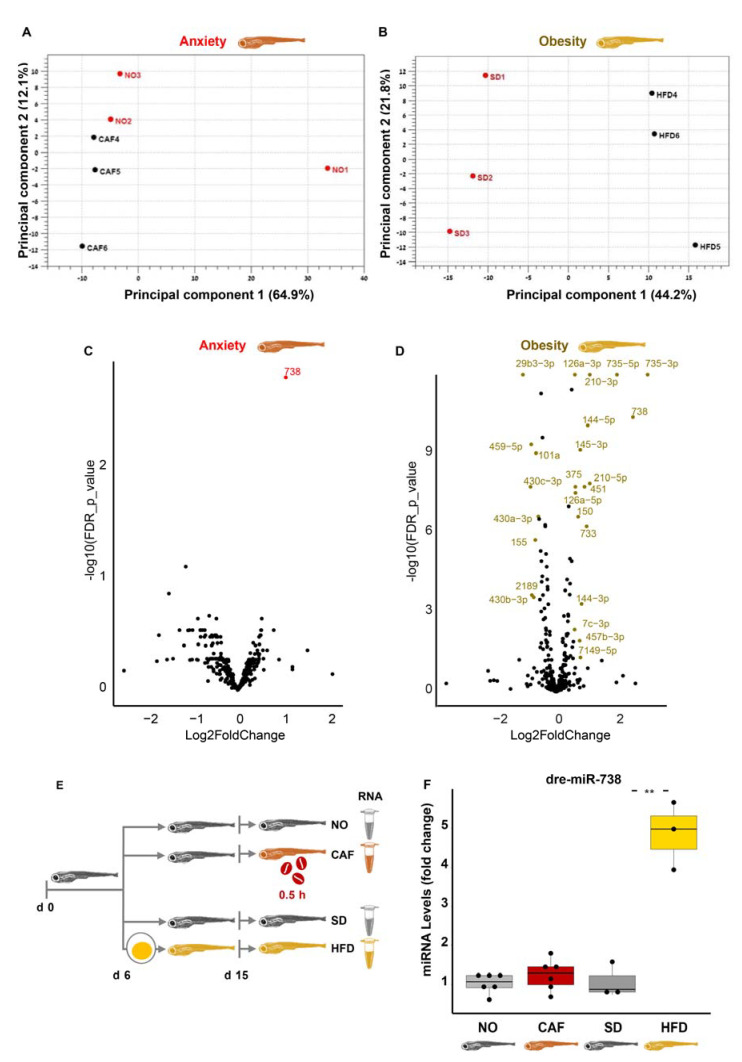
Null involvement of microRNAs (miRNAs) in the anxiety and obesity link in zebrafish larvae. (**A**,**B**) PCA graphs of anxiety and obesity, respectively. (**C**) Volcano plot of anxiety model. Red points: DE in the anxiety model, FDR < 0.05, and absolute log2 fold change > 0.58 (>1.5 and <0.67 fold change). The “dre-miR” prefix has been removed from miRNA labels to enable better visualization. (**D**) Volcano plot of obesity model. Gold points: DE in the obesity model, FDR < 0.05, and absolute log2 fold change > 0.58 (>1.5 and <0.67 fold change). (**E**) Experimental design: zebrafish larvae (6 days post-fertilization (dpf)) were fed either the standard diet (SD) or high-fat diet (HFD). At age 15 dpf, a group of the standard diet-fed larvae were further divided into caffeine-exposed larvae (CAF) and the controls (NO). Following caffeine exposure (0.5 h) of the CAF larvae, larvae of all treatments were snap-frozen until RNA extraction for RNA-seq. (**F**) qRT-PCR of dre-miR-738 in the anxiety and obesity models, Student’s *t*-test, *n* = 3–8 (10–30 larvae/replicate, and 1–3 independent experiments, where RNA of one of the experiments had been used in RNA-seq. The reference was the average of three miRNAs: dre-miR-let7a, dre-miR-125b-5p, and dre-miR-26a-5p. **—*p* < 0.01.

PCA analysis showed distinct miRNA clustering profiles in the obesity and anxiety models (Figure 4A,B), differing from the poly(A)+ RNAs, although they originated from the same RNA samples. Only one miRNA, *dre-miR-738*, was DE in the anxiety model (FDR < 0.05, fold change > 1.5 or <0.67) (Figure 4C). It was also upregulated in the obesity model, in which 24 other DE miRNAs were observed (FDR < 0.05, fold change > 1.5 or <0.67), and this upregulation was validated by qRT-PCR in the obesity but not the anxiety model (Figure 4D,F). In short, none of the significant and validated DE miRNAs in anxiety were also DE in obesity, contrasting adult mammals, where a meta-analysis indicated miRNA participation in the link between anxiety and metabolic syndrome [12].

### 3.9. Larval tRFs Were Upregulated in Anxiety and Downregulated in Obesity

**Figure 5:**
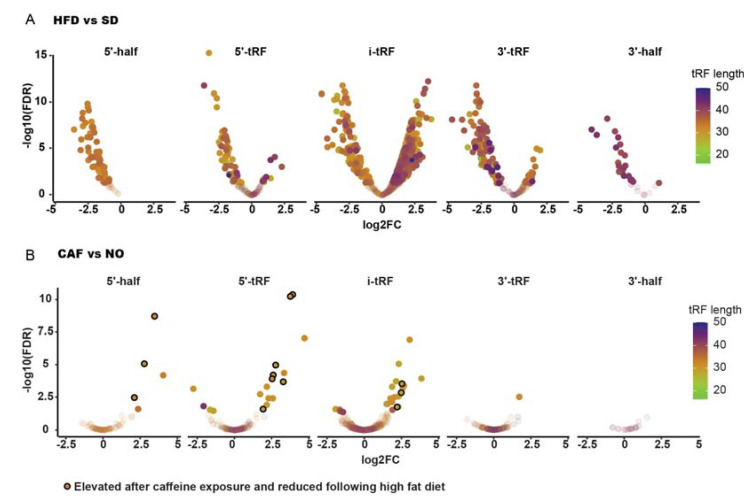
Transfer RNA fragments (tRFs), as well, are inversely regulated in anxiety and obesity. (**A**) HFD vs. SD. (**B**) CAF vs. NO. The encircled dots are the 13 tRFs that are upregulated in anxiety and downregulated in obesity.

Recent studies reveal that tRFs are regulatory tRNA-derived molecules that in certain neuro-pathologies may replace miRNAs in the control of immune system homeostasis [48,49]. Therefore, DE tRFs were sought, as well. Only 29–32 nts nuclear-derived tRFs were detected. Most DE tRFs (thirteen, eight of which were derived from Glu tRNA, excluding the option of coincidental change) were upregulated in anxiety and/or downregulated in obesity (Figure 5, Table 1), similar to the immune system-related inverse regulation phenomenon observed for poly(A)+ RNAs. Interestingly, three of the inversely regulated tRFs belong to the 5′-half tRF type, which are related to immune system reactions [48] and have been found to function in neurological disorders [50], tentatively implying their possible role in anxiety and obesity.

### 3.10. In Larvae, Obesity Fails to Accompany Anxiety, and Vice Versa

**Figure 6:**
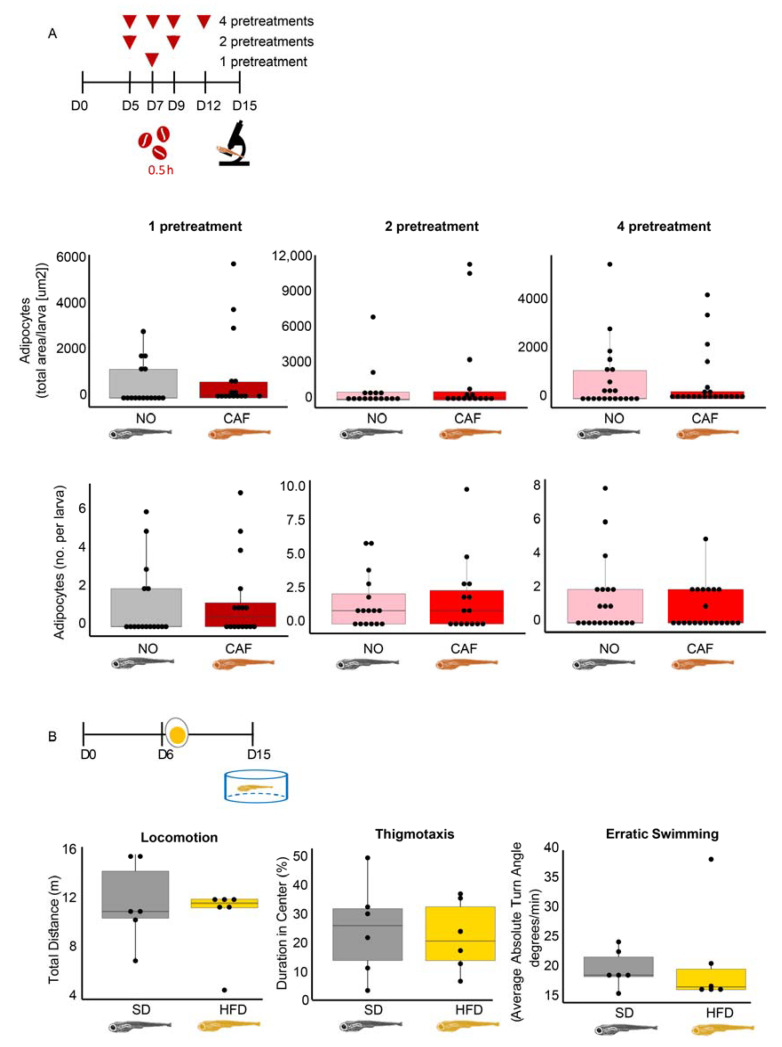
Obese larvae failed to present anxiety-like behavior and vice versa. (**A**) The timeline and results demonstrating the effect of anxiety (caffeine pretreatments (100 mg/L for 0.5 h)) on obesity (adipocyte size and number in the abdominal area of the larvae), *n* = 15–21. (**B**) The parallel effect of obesity (egg yolk solution-based HFD, 10 days) on anxiety-like behavior (thigmotaxis and erratic swimming), *n* = 6. Note the absence of apparent links between the two impacts.

When adult zebrafish become obese, they acquire anxiety-like behavior [13,14]. To examine if this occurs in larvae, we fed the larvae an HFD (for 10 days, as in the obesity model) and tested their anxiety-like behavior when they were 15 dpf. We also tested the effect of anxiety on obesity. Neither one (on 7 dpf), two (on 5 and 9 dpf), or four (on 5, 7, 9, and 12 dpf) high-dose caffeine pretreatments (0.5 h, as in the larval anxiety model) increased abdominal adiposity in the 15 dpf larvae (Figure 6A). Most importantly, no difference in thigmotaxis or erratic swimming was found between SD- and HFD-fed larvae (Figure 6B). We conclude that unlike adult fish, and although larvae have immune-related inverse regulation between anxiety and obesity, larval obesity does not seem to lead to anxiety. The apparent lack of an anxiety–obesity link in zebrafish larvae tentatively indicates that the positive link between anxiety and metabolic disorders in vertebrates may develop with age.

### 3.11. Age and Sex Both Seem to Affect Immune System-Linked mRNA Transcripts

Given the apparently inverse regulation between anxiety and obesity in fish larvae, we tested if this regulation pattern is maintained with age and in both sexes or if it is particular to the larval developmental stage. For this purpose, we used an accepted anxiety model for adult zebrafish [60,61,86] to comparatively assess the levels of DE transcripts that had been upregulated in our anxiety larval model and downregulated in our obesity larval model. This adult anxiety model was validated in middle-aged adult fish in three independent experiments (each with male and female fish). Notably, caffeine-exposed fish (of both sexes) swam significantly less to the top half of the tank, spent less time in the top half, and remained closer to the bottom of the tank than those that were not exposed to caffeine (Figure S5). Such behavior is characteristic of anxiety in adult zebrafish [60,61,86].

**Figure 7:**
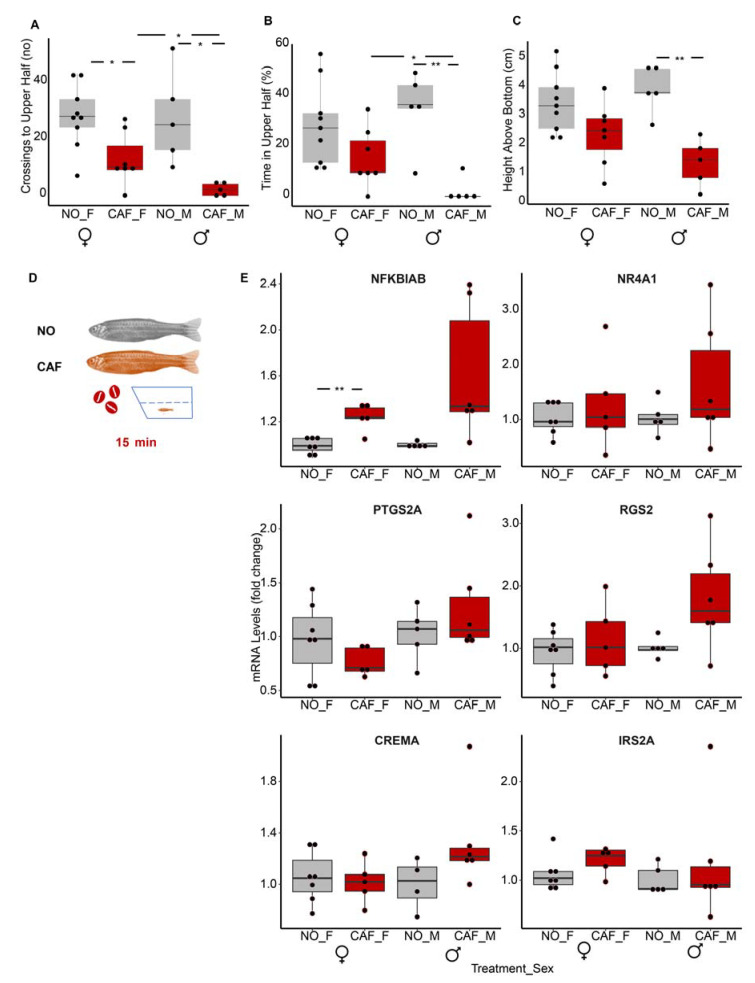
The nuclear factor kappa-light-chain-enhancer of activated B cells (NFκB) inhibitor (nfkbiab) transcript was upregulated in whole bodies of anxiety-induced middle-aged adult males and females. Anxiety-like behavioral tests: (**A**) crossings to upper half of tank, (**B**) percent of the time in the upper half of tank (**C**) height above the bottom of the tank. NO F, CAF F—non-treated and caffeine-exposed females; NO M, CAF M—non-treated and caffeine-exposed males. *n* = 5 male and *n*= 7–9 female fish in three independent experiments (each experiment had both males and females). Student’s *t*-test, *—*p* < 0.05, **—*p* < 0.01. (**D**) Scheme showing that individual fish were exposed to water or water containing caffeine in the tank for 15 min, during which movements were recorded. NO—no treatment; CAF—caffeine exposure. (**E**) qRT-PCR results: *n*= 4–6 male fish and 5–7 females in each treatment; two independent experiments, with males and females in each. The reference genes were actb2 and eef1a1l1. Student’s *t*-test.

Results were more pronounced in males. Although both caffeine-exposed females and males crossed to the top half of the tank significantly more than the controls (Figure 7A), only males exposed to caffeine spent significantly more time at the bottom and remained closer to the bottom of the tank than the male controls (Figure 7B,C). Despite the clear anxiety-like behavior of the adult zebrafish, only one of the six immune system-related DE transcripts found in larvae, nfkbiab, was DE in adults. It increased in the whole-body tissues of females by 24% (*p* = 0.006) and males by 61% (*p* = 0.050) (Figure 7D); this is less than in the larvae, in which its level doubled following caffeine exposure. None of the other mRNAs tested were upregulated in the whole bodies of adults.

Since in a whole-body measurement, an increased level of mRNA in one organ may cancel out the decreased level of the same mRNA in another, isolated organs of the above middle-aged fish, i.e., brain, liver, intestine, and a segment preceding the tail fin (consisting of skin, muscle, and bone), were also assessed for the expression of genes that had been upregulated in the anxiety larval model. In contrast to what was observed in the larvae, none of these genes were upregulated in the tested organs of adult fish exposed to caffeine (Figure S6). Rather, nr4a1 was downregulated in the female brain, contrasting its upregulation in larvae. Ptgs2a (Cox2a) and nr4a1 were not expressed in the livers of these fish. Thus, the upregulation initiated in anxiety in zebrafish larvae did not persist in middle-aged adults, and anxiety-like behavior resulting from caffeine exposure seems to differ among sexes; it was more pronounced in adult males than females. This may result from the inherently higher anxiety level in adult zebrafish females compared to males [87].

### 3.12. LncRNA Profiles Changed with Age and Sex

Similar to DE mRNAs that were apparently affected by age, lncRNAs were also influenced by age. Seven lincRNAs were upregulated by about two-fold and one processed transcript was downregulated by about 30% in the larval anxiety model (mentioned above). However, none of these were up- or downregulated in whole bodies of middle-aged zebrafish with induced anxiety, although we observed higher levels of these lincRNAs in males compared to females. Cautiously, we note that this difference might be attributed to the eggs in the abdomen of females, which might not express these lincRNAs (Figure S7).

The levels of these seven lincRNAs were also assessed in separate organs of adult male zebrafish, i.e., brain, intestine, liver, testes, and a portion of the tail, consisting of muscle, skin, and bone. As was found in whole-body middle-aged fish, caffeine had no significant effect on their levels in the various organs (Figure S8). DE was not apparent, conceivably because of a high variation between samples. Moreover, although all seven lincRNAs were found in the muscle/skin/bone sample and the brain, we could not detect *FO681323.202*, *CABZ01048956-203*, and *CR926130-201* in the liver and intestine. Disassociation analysis identified two distinct variants of *FO834828-201*, depending on the tissue; in the muscle, skin, and bone samples, it had a melting temperature of ~75, while in the other organs tested, it had a melting temperature of ~84. While these lincRNAs also require further analyses, their distinct expression profiles seem to support the notion of age-dependent differences in the links between anxiety and obesity.

### 3.13. Inversely Regulated Inflammation-Related DE Transcripts Differed between Zebrafish Adults and Larvae

**Figure 8:**
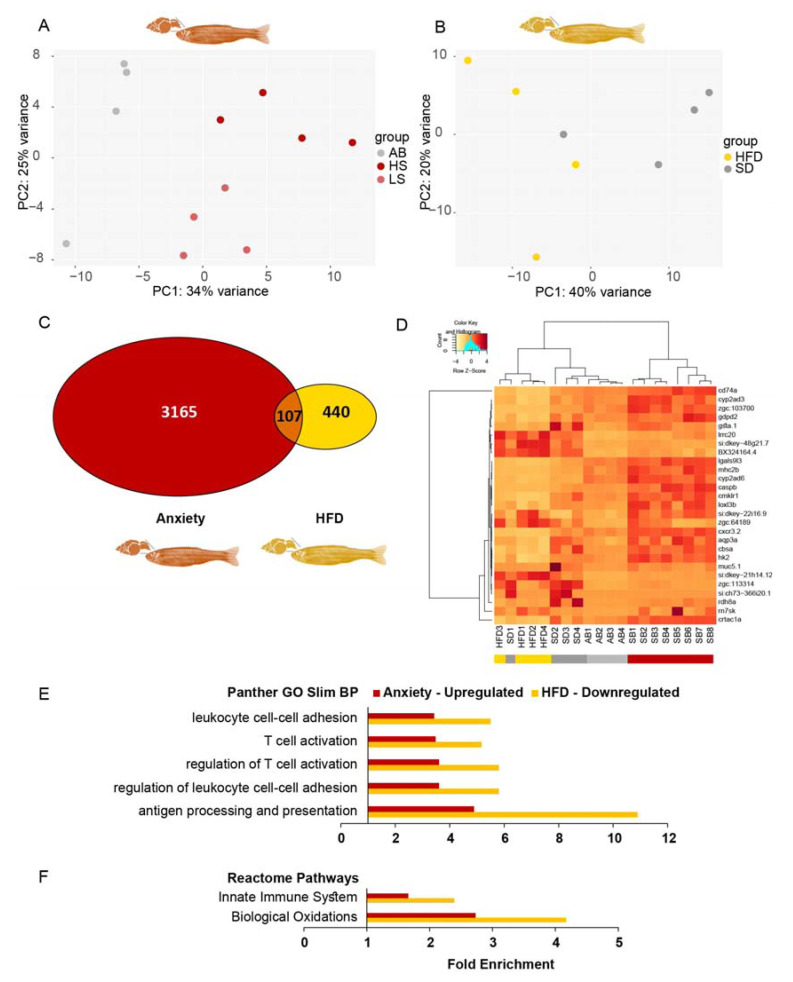
Immune system-related gene ontology (GO) terms are enriched among transcripts increased in anxiety and decreased under high-fat diet (HFD) conditions in adult zebrafish. (**A**) PCA of transcripts from young adult zebrafish brains: AB—low anxiety; SB (HS and LS)—high anxiety. (**B**) PCA of transcripts from telencephalons of young adult zebrafish fed HFD or SD. (**C**) A total of 107 transcripts were commonly differentially expressed (DE) (padj < 0.05) in anxiety (red) and HFD (yellow) in adult zebrafish. (**D**) Heatmap of the 27 transcripts upregulated (>1.5) in anxiety among the DE (padj < 0.05) intersecting genes in zebrafish adults; AB—low anxiety, SB—high anxiety. (**E**) Overrepresented Panther GO-slim biological processes (BP) common in upregulated anxiety and downregulated HFD genes in zebrafish adults. (**F**) Overrepresented Reactome Pathways common to upregulated anxiety and downregulated HFD genes in zebrafish adults. Reference transcripts were all found in the Danio rerio database, Fisher’s exact test, and correction by FDR < 0.05.

We analyzed two young adult (3 months post-fertilization) zebrafish RNA-seq datasets, one from whole brains of inherently anxious adult zebrafish (LSB and HSB strains that we termed SB) versus less anxious ones (AB strain) [71,72], and the other from telencephalons of HFD- versus SD-fed adult zebrafish (that were fed HFD for 11 weeks and tended to be more obese than SD-fed fish) [73]. We applied the same pipeline used for the larval data to these adult datasets. The individual transcript profiles in both datasets clustered satisfactorily according to treatment (Figure 8A,B). There were 3272 anxiety-related DE genes (padj < 0.05) compared to only 547 HFD-related DE transcripts (padj < 0.05). The larger number of anxiety-DE genes could be strain-related, with only part of the genes associated with anxiety, and the small number of HFD-DE genes was attributed to them being brain-derived rather than liver or adipose tissue sources.

Similar to larvae, more transcripts were upregulated (850, >1.5-fold) than downregulated in anxiety (563, <0.67-fold), and more transcripts were downregulated (378) than upregulated in HFD-fed fish (62). Only 107 transcripts were DE and common to both the anxious and HFD-fed fish (padj < 0.05) (Figure 8C), with only 2 genes appearing also among the 231 anxiety–obesity DE genes in larvae (*zgc:162730* and *ets2* (v-ets avian erythroblastosis virus E26 oncogene homolog 2)). Most unexpectedly, 20 (74%) of the 27 transcripts upregulated (>1.5-fold) in anxious adult brains were present among those downregulated (<0.67-fold) in the HFD-fed adult zebrafish (reflected in the heatmap, Figure 8D). Notably, these 20 genes were related to the immune system, e.g., chemokine-like receptor 1 (*cmklr1*); chemokine (C-X-C motif) receptor 3, tandem duplicate 2 (*cxcr3.2*); CD74 molecule, major histocompatibility complex, and class II invariant chain a (*cd74a*); caspase b (*caspb*); lectin, galactoside-binding, soluble, 9 (galectin 9)-like 3 (*lgals9l3*); and major histocompatibility complex class II integral membrane beta chain gene (*mhc2b*).

To further assess the function of the DE transcripts in anxious and HFD-fed adult zebrafish, we examined the GO terms of the upregulated and downregulated DE transcripts (padj < 0.05) in each condition using Panther. Common GO terms were found only among the upregulated transcripts of the anxious adult zebrafish and the downregulated ones in the HFD-fed adult zebrafish, and these terms were almost exclusively related to the immune system. GO-slim overrepresented biological process (BP) terms included “leukocyte cell-cell adhesion”, “T cell activation”, and “antigen processing and presentation”, as well as Reactome Pathways, such as the “innate immune system” and “biological oxidations” (Figure 8E,F). Thus, conceptually, inverse regulation of immune-related genes between anxiety and obesity initiates in larvae is sustained in adult zebrafish but is based on different sets of genes, probably since larvae only have the innate immune system, whereas the adults have the innate and adaptive immune systems [31].

## 4. Discussion

Many studies have sought mechanisms linking anxiety disorders and metabolic syndrome/obesity [9,10,19,22,88,89,90,91]. However, to our knowledge, none have tested these links in a single study using unbiased transcriptomics to identify the underlying processes and the subsets of RNA transcripts involved. Our findings indicate correlatively that in zebrafish larvae, most of the DE transcripts demonstrated inverse regulation in anxiety and obesity, with anxious larvae presenting mostly upregulated transcripts, contrasting with a downregulation pattern in obese larvae. Moreover, gene ontogeny analysis showed that an overrepresented subset of these inversely regulated protein-coding genes participates in inflammation/immune system-related pathways. While this inverse regulation of immune genes in anxiety–obesity comparisons persisted in adult zebrafish according to our transcriptomic analysis, the genes inversely regulated in the adults differed from those in the larvae. These results raise the question whether this well-known link between anxiety and obesity is subject to age with an antagonistic pleiotropic pattern, and if HFD can protect from anxiety and if so, would such protection persist throughout life.

The long RNA sequencing of the larval anxiety and obesity models revealed that as many as 75% of the genes (including lincRNAs) common to both models that were upregulated in anxiety were also downregulated in obesity, and these inversely regulated DE genes were overrepresented in the following immune system-related pathways: (1) “CCKR signaling map (gastrin- and cholecystokinin-mediated regulation of cellular processes)”, (2) “inflammation mediated by chemokine and cytokine signaling pathway”, and (3) “interleukin signaling pathway”. Correspondingly, the downregulation of immune-related genes was revealed in transcriptomes of insulin-resistant zebrafish larvae [92].

Apart from the long RNA sequences, thirteen tRFs were discovered to be upregulated in anxiety and downregulated in obesity. These tRFs (29–32 nts) are probably too long to bind to the RNA-induced silencing complex (RISC) and, therefore, may not act as miRNAs, which are typically ~22 nts [93]; however, they may bind certain protein partners. Specifically, the inversely regulated tRFs include three 5′ tRF halves that have been implicated in immune-related functions [48] and may potentially participate in immune reactions related to anxiety and obesity. Of these three 5′ halves, *tRF-32-PSQP4PW3FJI01* was found to be upregulated in breast cancer tumors compared to adjacent normal tissue [94], *tRF-32-86V8WPMN1E8YN* was upregulated in human malignant mesothelioma vs. normal mesothelium [95], and *tRF-32-87R8WP9N1EWJM* was detected in blood cells [49]. Thus, tRFs may also take part in the immune system-related inverse regulation observed in anxiety and obesity.

Since our results implied that the inflammation-related pathways, present in the anxiety-induced larvae, were absent in the caffeine-induced anxious adult fish, it was important to perform analysis of RNA sequencing of anxious and obese adult zebrafish. Thus, we analyzed crude data transcript files (FASTQ RNA sequencing files) from two different research groups, at the risk of generating genetic background noise. One dataset was from whole brains of inherently anxious adult zebrafish versus less-anxious ones [71,72] and the other from telencephalons of HFD- versus SD-fed adult zebrafish (although the telencephalon of zebrafish differs morphologically from the homologous cerebrum in mammals; its mammalian counterparts have similar functions [33]). Surprisingly, similar to the larvae, 74% of the transcripts upregulated in anxious adults were present among those downregulated in the HFD-fed adult zebrafish, and these genes included immune-related genes and gene functions, distinct from those found in the larvae. In agreement with our findings in anxious adult zebrafish, mice lacking CD4+ T cells did not develop stress-induced anxiety, and CD4+ T cell-secreted xanthine and meningeal T cell-derived interferon-γ have been found to participate in murine anxiety-like behavior [96,97]. Furthermore, supporting the HFD/obesity-induced downregulation of immune system-related processes found in our study is the enrichment of the chemokine signaling pathway found among the decreased transcripts in HFD-fed mice cortices [98], and the significantly lower weight of HFD-fed rats thymuses compared to the controls [99]. Moreover, surgery-based weight loss in humans was characterized by the enrichment of antigen processing/presentation and interferon γ pathways in subcutaneous fat among the upregulated genes [100]. Gene ontology terms of one study presented conflicting findings; Stat5a/b knockout obese mouse hypothalamus-upregulated transcripts were associated with insulin stimulus, lipid and saccharide metabolic process, fat cell differentiation, and positive regulation of the adaptive and innate immune responses [101].

Inflammation has been associated with both obesity [80,102,103] and anxiety disorders [104,105] and has been studied as a possible link between both disorders [9,19,90,91], seemingly contrasting with our findings on inverse regulation between obesity and anxiety in fish larvae and adults. However, in many of the studies that demonstrated inflammation in both obese and anxious rodents, anti-inflammatory treatments only mitigated anxiety and inflammation but did not affect obesity. Diet-induced obese mice showed greater anxiety, more inflammation, and less insulin signaling in the nucleus accumbens than the controls; yet anti-inflammatory treatment diminished this anxiety and inflammation and improved insulin signaling but did not reverse HFD-induced obesity [89]. In leptin receptor-null obese mice (db/db vs. db/+), chronic food restriction or anti-inflammatory treatment reduced anxiety-like behavior and hippocampal and/or peripheral inflammation, but anti-inflammatory treatment did not reverse the obese state [90]. Moreover, acetylcholine (ACh), which is globally elevated in anxiety states [106], can block NFκB-mediated inflammation [12,107]; and transgenic excess of the acetylcholinesterase-targeting miR-132 [108], which exacerbates anxiety [109] by limiting acetylcholinesterase (which would elevate acetylcholine levels and further reduce inflammation), induces obesity [110]. Thus, anti-inflammatory treatment can reduce anxiety but not ameliorate obesity, which is compatible with our findings of lessened immune-related functions in HFD/obesity. Nevertheless, our results, revealing an overrepresentation of immune-related GO terms among the downregulated genes in obese larvae, do not necessarily imply reduced inflammation; since, among the genes validated by qRT-PCR (*ptgs2a*, *nfkbiab*, *crema*, *rgs2*, and *nr4a1*), most are able to suppress and/or induce inflammation, depending on the conditions [77,78,79,80,81,82,83]. Moreover, the overrepresented immune system-related GO terms among the downregulated genes in HFD-fed adult zebrafish belong to the telencephalon of the brain and not to the whole brain, adipose tissue, or liver, which if analyzed instead, may have led to a very different conclusion.

Although a high-fat diet and obesity are often reported to have adverse effects, inducing chronic low-grade inflammation [100,103] and increased risks of diabetes and cardiovascular diseases [8,103], recent studies report that HFDs and obesity can have a protective role, as implied by our study. Indeed, HFD/obesity downregulation of immune related genes could potentially normalize and thus protect zebrafish from an exaggerated immune function that can lead to allergies, autoimmune disease, and cancer [111]. For example, 12 weeks of HFD reduced anxiety-like behavior in rats compared to the controls [99] and protected mice (fed from 3 weeks old) from an increased level of anxiety-like behavior that accompanied chronic social stress [112]. Eight weeks of HFD reduced anxiety-like behavior generated by ovariectomy, regardless of the fat type, whether it is lard or fish oil [113]. In addition, HFD protected C57/Bl male mice from intraperitoneal-injected lipopolysaccharide-induced inflammation in the hypothalamus, although not in the hippocampus [114], and also protected against Alzheimer’s disease (AD) pathology in an AD mouse model [115].

HFD protection and its effects on diabetes are of special interest in the following cases. HFD-fed offspring of a rat model of type one diabetes, born to dams that were also fed HFD during pregnancy, had a lower incidence of diabetes than offspring fed a normal diet from dams fed a normal diet [116]. Moreover, twelve months of HFD in transgenic AD mice reduced blood–brain barrier leakage and brain atrophy, improved cognition, and returned levels of insulin receptor mRNA to that of the normal wild type mice [117]. Furthermore, adult offspring of AD or wild type mice fed an HFD only during their 3-week gestation had less memory decline, better synaptic function, and less tau [118,119]. Nevertheless, evidence exists that contradicts the positive effect of HFD on cognition in mice and zebrafish [73,98]. Yet, Western diet (high sucrose/HFD)-induced obese minipigs had higher memory scores than the lean Western diet controls [120].

HFD protection occurs in the following intriguing pathways and conditions, as well. In rats, short-term HFD reduced hemorrhagic shock-induced systemic inflammation via CCK receptor stimulation of the afferent vagal nerve and the binding of acetylcholine to nicotinic receptors on macrophages [121]. In mice, HFD-induced obesity decreased ventilator-induced lung injury, possibly by lowering the levels of matrix metalloproteinase (MMP) activity and soluble receptor for advanced glycation end-products (sRAGE, an epithelial stress marker) in obese mice compared to the controls [122]. In humans, overweightness or obesity were independently associated with lower 60-day in-hospital mortality [123]. Similarly, a meta-analysis and another extensive study (730 intensive care units in 84 countries) showed that obesity was correlated with decreased hospital mortality [124]. In summary, HFD and obesity can have protective effects. Our current findings in zebrafish hint that these protective effects of HFD and obesity may operate via immune pathways, exert homeostatic effects on the anxiety-upregulated immunity system-associated genes, and relate to the HFD protection reported by others.

The results of this study are correlative, which is a major limitation. However, understanding this inverse regulation may aid in further elucidating the mechanisms of metabolic and anxiety disorders. Other limitations relate to the larval transcriptomic data, which was derived from only one obesity model, in which the larvae were fed a short termed 10-day HFD vs. SD. Additional data from a longer-termed HFD and/or a Western diet may differ in the transcriptomic effects of obesity. In addition, we only sequenced larvae from one anxiety model. Optional larval anxiety models include recent transgenic models, e.g., (vesicular monoamine transporter 2 knockdown [125], a neurexin2aa deficiency [126], serpini 1 knockout [127], and brain-derived neurotropic factor loss [128]); however, there is no one known gene that causes anxiety [16], and, therefore, no one transgenic anxiety model. There are two accepted zebrafish anxiety models; one utilizes the acute exposure of alarm pheromone (collected from damaged epidermal cells of zebrafish) [34,129], while the other employs acute caffeine exposure to induce anxiety-like behavior in zebrafish [59,60,61]. We chose the latter. It would, however, be very interesting to obtain RNA-seq data from the very recently reported chronic unpredictable stress larval model [130].

One may argue that caffeine exposure or consumption, which not only induces anxiety-like behavior in zebrafish [59,60,61] but is also a known anxiogenic agent in humans [131] and mice [132], can be anti-obesogenic. Supporting this notion, in non-obese humans, caffeine consumption (4 or 8 mg/kg body weight, equivalent to 2–3 or 5–6 cups of coffee, respectively) increased the metabolic rate and plasma-free fatty acid levels relative to the controls [133]. However, in obese people on a low-calorie diet, caffeine consumption, even relatively high amounts (600 mg/day), has not been effective in promoting weight loss [134]. In overfed zebrafish larvae, chronic low dose caffeine exposure reduced the levels of hepatic lipogenic factors and inflammation-related genes and limited hepatic steatosis [135]. Such low doses improve attention in zebrafish but do not induce anxiety [136]. In contrast, high doses of caffeine generate nervousness in humans [131] and anxiety-like behavior in both adult zebrafish [60,61] and zebrafish larvae [59], which is compatible with our current findings. Additionally, our research supports the use of caffeine in the anxiety model, since in both caffeine-induced anxiety-like behaved zebrafish larvae and inherently anxious adult zebrafish, we found the upregulation of immune-related genes.

Based on the above, immune system-related processes seem to be inversely regulated in anxiety and HFD in both zebrafish larvae and adults. However, the specific genes and their functions in larvae differ from adults. This raises the speculation that the set of inverse-regulated immune genes in adults may associate with the positive anxiety–obesity link (meaning that obesity leads to anxiety) that has been determined empirically in adult rodents [9,10,89], adult humans [7,91], and adult zebrafish [13,14]. Such a positive anxiety–obesity link was revealed to be lacking in zebrafish larvae, which is in agreement with another recent study [13]. This positive anxiety–obesity link is correlated with a different set of inversely regulated immune genes in adult zebrafish compared to larvae. As larvae only have an innate immune system, whereas adult zebrafish have both innate and adaptive immune systems [31], it may be possible that adaptive immune reactions are important for the development of the positive anxiety–obesity links.

At a more far-reaching level, we hypothesize that the development-related link between anxiety and obesity, which involves distinct immune system elements at different ages, indicates a possible antagonistic pleiotropic pattern of change in the immune system response. Antagonistic pleiotropy refers to genes that impart traits, which benefit reproduction and fitness under one condition (e.g., young age), but may impede them under another (e.g., older age) [137]. The inversely regulated immune system-related genes found in anxiety and obesity in zebrafish may improve survival in young organisms and hinder them later in life. HFD/obesity can be advantageous when food is scarce and for young larvae, which may have a high daily energy expenditure if similar to human infants [138]. However, extreme anxiety or maternal anxiety disorders are harmful [139]. The inversely regulated anxiety–obesity immune system genes are compatible with the lack of an anxiety–obesity link during early development. Therefore, obese larvae will not develop anxiety, conferring a survival advantage, whereas, in adult zebrafish, the anxiety–obesity immune system correlates with an inversely regulated anxiety–obesity link, in which obesity leads to anxiety [13,14], which is disadvantageous. This link may be due to the need to avoid disrupted physiological homeostasis, causing or preventing age-related diseases. Therefore, the immune system-based link between anxiety and obesity may have an antagonistic pleiotropic pattern.

## 5. Conclusions

In conclusion, our findings indicate an inverse regulation between anxiety and obesity, with the upregulation of most transcripts in anxious larvae, contrasting with the downregulation of these same transcripts in obese larvae. Furthermore, immune system-related pathways were overrepresented in both anxiety and obesity in zebrafish larvae compared to under normal conditions. This anxiety–obesity inverse regulation was also present in adult zebrafish. However, the genes related to this inverse regulation in adult zebrafish differed from those in larvae. This gene difference may be tentatively attributed to the presence in adults of the adaptive immune system, which is lacking in larvae. Since HFD did not lead to anxiety-like behavior in larvae, contrasting with what is known in adult zebrafish, these results hint at an antagonistic pleiotropic pattern regarding the inter-relation between anxiety and obesity.

## Figures and Tables

**Table 1:** Inversely regulated transfer RNA fragments (tRFs) are upregulated in anxiety and downregulated in obesity.

tRF	Sequence	Type	tRNA
**tRF-29-JQR7F8YKIRJZ**	CATATGGTCTAGCGGTTAGGATTCCTGGT	i-tRF	Glu
**tRF-30-FP18LPMBQ4NK**	AGCAGAGTGGCGCAGCGGAAGCGTGCTGGG	5′-tRF	Met
**tRF-30-KQI34JRX6N38**	CCCATATGGTCTAGCGGTTAGGATTCCTGG	i-tRF	Glu
**tRF-30-KY7343RX6NMH**	CCCTGGTGGTCTAGTGGTTAGGATTCGGCG	i-tRF	Glu
**tRF-30-PIR8YP9LON4V**	GCACTGGTGGTTCAGTGGTAGAATTCTCGC	5′-tRF	Val
**tRF-31-86V8WPMN1E8Y0**	TCCCATATGGTCTAGCGGTTAGGATTCCTGG	5′-tRF	Glu
**tRF-31-87R8WP9I1EWJ0**	TCCCTGGTGGTCTAGTGGCTAGGATTCGGCG	5′-tRF	Glu
**tRF-31-87R8WP9N1EWJ0**	TCCCTGGTGGTCTAGTGGTTAGGATTCGGCG	5′-tRF	Glu
**tRF-31-PSQP4PW3FJI0B**	GCCCGGCTAGCTCAGTCGGTAGAGCATGAGA	5′-tRF	Lys
**tRF-31-PW5SVP9N15WV0**	GCCGTGATCGTATAGTGGTTAGTACTCTGCG	5′-tRF	His
**tRF-32-86V8WPMN1E8YN**	TCCCATATGGTCTAGCGGTTAGGATTCCTGGT	5′-half	Glu
**tRF-32-87R8WP9N1EWJM**	TCCCTGGTGGTCTAGTGGTTAGGATTCGGCGC	5′-half	Glu
**tRF-32-PSQP4PW3FJI01**	GCCCGGCTAGCTCAGTCGGTAGAGCATGAGAC	5′-half	Lys

## Data Availability

RNA-seq poly(A)+ dataset: Gene Expression Omnibus, SuperSeries GSE207550 (https://www.ncbi.nlm.nih.gov/geo/query/acc.cgi?acc=GSE207550)/SubSeries GSE207457 (https://www.ncbi.nlm.nih.gov/geo/query/acc.cgi?acc=GSE207457). Small RNA-seq dataset: Gene Expression Omnibus, SuperSeries GSE207550 (https://www.ncbi.nlm.nih.gov/geo/query/acc.cgi?acc=GSE207550)/SubSeries GSE207549 (https://www.ncbi.nlm.nih.gov/geo/query/acc.cgi?acc=GSE207549).

## Supplementary Materials

The following supporting information can be downloaded at https://www.mdpi.com/article/10.3390/cells12131794/s1, Supplementary File (Figure S1: Anxiety and Obesity Models; Figure S2: Panther Gene ontology (GO) terms that were overrepresented among anxiety and obesity DE genes; Figure S3: Panther Gene ontology (GO) terms overrepresented among non-immune genes of the anxiety-obesity inversely regulated genes; Figure S4: Both RNA-seq and qRT-PCR validated processed transcript lncRNA transcript, si:dkey-7c18.24-203; Figure S5: Caffeine exposure induced strong anxiety-like behavior in adult zebrafish; Figure S6: Transcripts upregulated in caffeine-treated larvae were not upregulated in organs of middle-aged caffeine-treated adult males and females; Figure S7: lncRNAs were more frequently expressed in males than in females; Figure S8: Certain lincRNAs were expressed in all tested organs of adult zebrafish; Table S1: Primer sequences; Table S2: RNA transcript types among the isoforms identified in poly(A)+ RNA-seq of zebrafish larvae; Table S3: Characteristics of lncRNAs; Table S4: LincRNA neighboring mRNAs).
