# Supplementary material for: Inversely Regulated Inflammation-Related Processes Mediate Anxiety–Obesity Links in Zebrafish Larvae and Adults
Source: Cells. 2023 Jul 6;12(13):1794. doi: 10.3390/cells12131794 (PMC10341043; doi:10.3390/cells12131794)
Supplement: Supplementary file 1 [file cells-12-01794-s001.zip › cells-2478317-supplementary.pdf]

Supplementary File – Yehuda et al.

Table of Contents

Table S1. Primer sequences 2

.... 4

Figure S1. Anxiety and Obesity Models.. 5

Table S2. RNA transcript types among the isoforms identified in poly(A)+ RNA-seq of zebrafish larvae 6

Figure S2. Panther Gene ontology (GO) terms that were overrepresented among anxiety and obesity DE genes 7

Figure S3. Panther Gene ontology (GO) terms overrepresented among non-immune genes of the anxiety-obesity inversely regulated genes. 8

Figure S4. Both RNA-seq and qRT-PCR validated processed transcript lncRNA transcript, si:dkey-7c18.24-203. 9

Table S3. Characteristics of lncRNAs 10

Table S4. LincRNA neighboring mRNAs.. 11

Figure S5. – Caffeine exposure induced strong anxiety-like behavior in adult zebrafish. 13

Figure S6. Transcripts upregulated in caffeine-treated larvae were not upregulated in organs of middle-aged caffeine-treated adult males and females. 15

Figure S7. lncRNAs were more frequently expressed in males than in females. 16

Figure S8. Certain lincRNAs were expressed in all tested organs of adult zebrafish. 17

Table S1. Primer sequences

Gene/Isoform	Primer Sequence (5'-3')	
	**Forward**	**Reverse**
**nfkbiab**	GTCGCCATCCAGGGTTACTT	GGCCGTTACACTGCTCCTGT
**crema**	TGATGAAGAACAGGGAGGCAG	TGAGCAGTGTTTGTGAGGGTT
**rgs2**	GCTAAGCAGCAAATGTGGAC	AGGCCAACCAGAAGTCAAGA
**irs2a**	GCAACCACTGTACAAGATTGAGC	AGGTGCAAAGGTCACGGTTC
**nr4a1**	GGGGATGGTGAAGGAAGTTGT	CTGGCAGGGTTTGAGTCGAT
**cox2a**	CTGGGTCATGGAGTGGATCTG	TTCCGGGATATGAGGAGGGT
**actb2**	CCAGCCATCCTTCTTGGGTAT	CTTCATTGTGCTAGGGGCCA
**eef1a1l1**	ACCTACCCTCCTCTTGGTCG	GGAACGGTGTGATTGAGGGAA
**BX908796.4-201**	GTAGAGCTCCTCCGAAACATCA	TGCCCGACTAAGACAAACCA
**BX465834.1-204**	CACGTCTCCAATGTGAACGC	TCAGTTTTCATCTGCTCTGTGG
**CABZ01048956.1-203**	CCAGAGTTCTGCTCTTTGGC	GGTGCTTTCTGTCACTTGGC
**FO834828.2-201**	CAGCAGAAGGAGGTGGTGTT	TGTGTGGTTGGTGCATCAGT
**FO681323.1-202**	CTTCAACCAAATTGCTTTCTTGAGC	TGCATTTGTGCTGTTGCCTG
**FO904966.2-201**	GTGTCAGTGGTGCTGCCTTT	CACTACCAATAGCACCAGCG
**AL954191.1-201**	CCAGACAACCTCTTCTCGTG	TGTCAGTGTCCAGGTTTCCT
**CR926130.2-201**	TGATTCTTAAAGGATTCCGTCGT	GTTCTGAGACTGAACTGTCATTACT
**CR926130.2-202**	TCTGTGTTTTCCTTCTAGTCCTGG	TGTGAGACTGAACTTTCATTACTGG
**BX649294.1-203**	CCGCTTTCCATCCCAAGAGT	TCCTTGTTGTCCATCTCCGC
**zgc:101716-202**	TTGGCCAGAGGACAGACGTA	AAGCTGGTGGCCAGATTCAG
**si:dkey-7c18.24-203**	TGTTCACTCCCTTTTTGGACA	TGGGTACATCAGGATTGAGAAAT
**gpr108-201**	AGTGGCAGTGGTGTCAAGAG	CCCGACTCGGTTACAACTTCA
**hdac9b-203**	GTCTCCATCGCTGCCCAATA	TGCATTCTATTTGTGTGCATCCC
**BX537288.2-204**	GATGCGCTAATGGACGACAC	CAAGCTCCTCAAGACACCCA
**dre-miR-738**	CTACGGCCCGCGT	GTCCAGTTTTTTTTTTTTTTTGAGGT

**dre-miR-26a-5p**	GCAGTTCAAGTAATCCAGGATAG	GGTCCAGTTTTTTTTTTTTTTTAGC
**dre-miR-125b-5p**	GCAGTCCCTGAGACCCT	CCAGTTTTTTTTTTTTTTTCACAAGT
**dre-let-7a**	GCAGTGAGGTAGTAGGTTG	GGTCCAGTTTTTTTTTTTTTTTAACTATAC

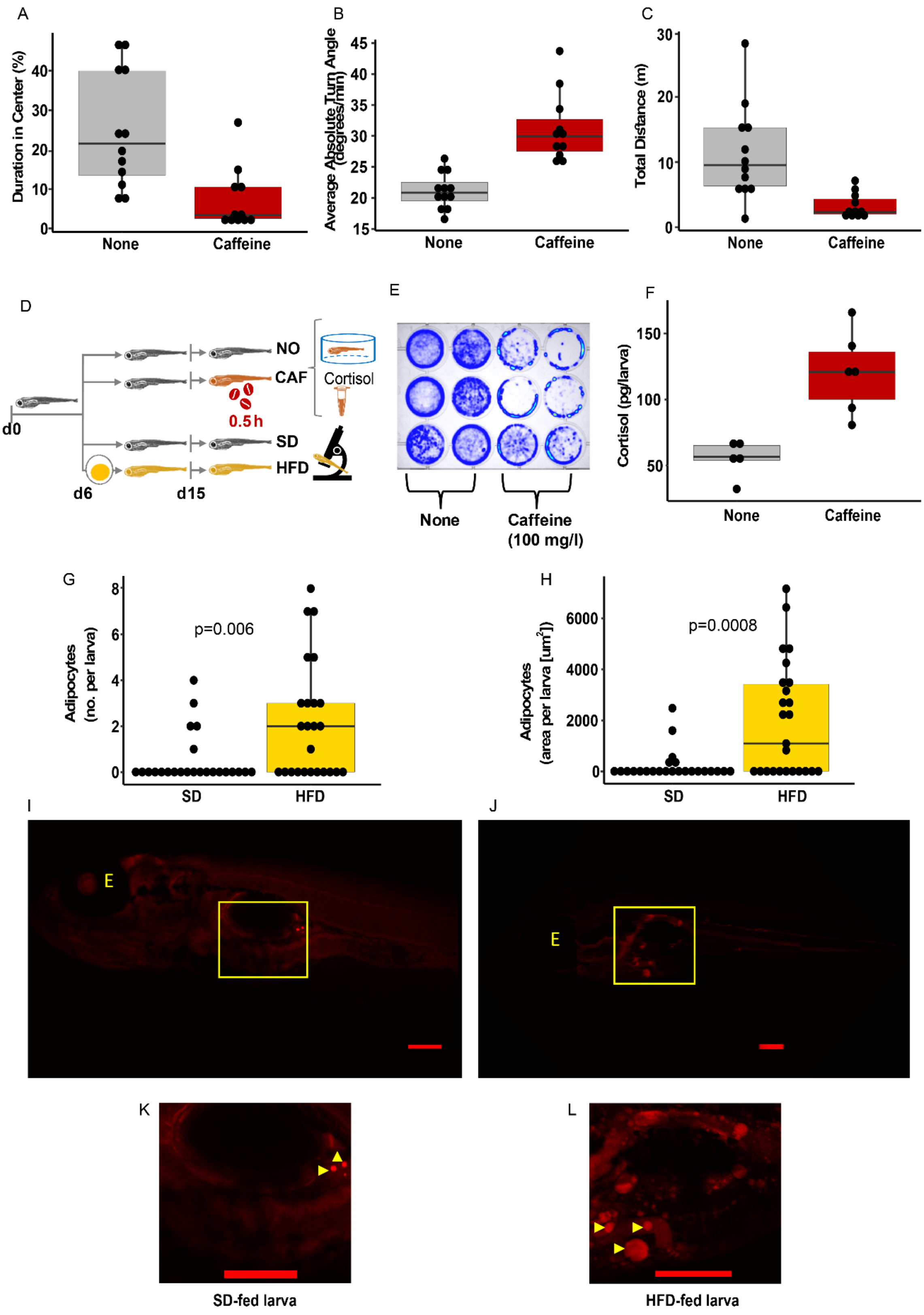
A
Duration in Center (%)
None
Caffeine
B
Average Absolute Turn Angle (degrees/min)
None
Caffeine
C
Total Distance (m)
None
Caffeine
D
NO
CAF
Cortisol
0.5 h
SD
HFD
d0
d6
d15
E
None
Caffeine (100 mg/l)
F
Cortisol (pg/larva)
None
Caffeine
G
Adipocytes (no. per larva)
p=0.006
SD
HFD
H
Adipocytes (area per larva [um²])
p=0.0008
SD
HFD
I
E
J
E
K
SD-fed larva
L
HFD-fed larva

Figure S1. Anxiety and Obesity Models - Behavior tests, thigmotaxis (A) and erratic swimming (B), indicate anxiety-like behavior, and locomotion (C) is the distance traveled, in two individual experiments, n=11-12, Welch Two Sample t-Test - p<0.003, Wilcoxon Test – p<0.002. Experimental design - zebrafish larvae (6 dpf) were fed either standard diet (SD) or high fat diet (HFD, based on hardboiled egg yolk solution). At age 15 dpf, a group of the standard diet fed larvae were further divided into caffeine-exposed larvae (CAF) and their controls (NO). Following caffeine exposure (0.5 h) of the CAF larvae, larvae of the anxiety model were tested for anxiety-like behavior or snap frozen for cortisol tests. Obesity model larvae were stained and histologically assessed for abdominal adipocytes on day 16 (D). Tracks of larvae (E): Each well contained a single larva whose position is represented by a heatmap (colors signify the frequency of a specific position, from blue the least, to red color the most). Whole body cortisol levels (F) are from two individual experiments, n=5-6, replicate = pool of 30 larvae, Welch Two Sample t-Test – p<0.003, Wilcoxon Test – p<0.005. Adipocyte number per larva (G) and adipocyte total area per larva (H) are from two independent experiments, n= 24-25, Welch Two Sample t-Test. The above pictures represent typical adipocyte-bearing larvae from each diet group, SD (I) and HFD (J), with the anterior on the left. Adipocytes contain lipid droplets stained red with Nile Red (I-L). E – Eye, SB – swim bladder in the peritoneum. An amplification of the area in the yellow square of (I) is in (K) and of that in (J) is in (L). Yellow arrowheads point to lipid droplets (presumed to be adipocytes). Scale bar = 200 µm.

Table S2. RNA transcript types among the isoforms identified in poly(A)+ RNA-seq of zebrafish larvae

RNA Transcript Type	Transcript numbers
**Protein-coding***	41203
**Retained intron**	3107
**Processed transcript**	2841
**LincRNA**	1901
**rRNA**	895
**Antisense**	754
**Nonsense-mediated-decay**	560
**Sense intronic**	54
**Sense overlapping**	9
**Other noncoding RNAs**	737
**Total**	**52061**

*Protein coding-gene/transcipt that contains an open reading frame (ORF); Retained intron- an alternatively spliced transcript believed to contain intronic sequence relative to other, coding transcripts of the same gene; Processed transcript- gene/transcript that lacks an open reading frame (ORF); LincRNA- transcripts that are long intergenic non-coding RNA; rRNA-ribosomal RNA; Antisense- transcripts that overlap the genomic span (i.e. exon or introns) of a protein-coding locus on the opposite strand; Sense intronic- a long non-coding transcript in introns of a coding gene that does not overlap any exons; Sense overlapping-a long non-coding transcript that contains a coding gene in its intron on the same strand. (http://www.ensembl.org/info/genome/genebuild/biotypes.html)

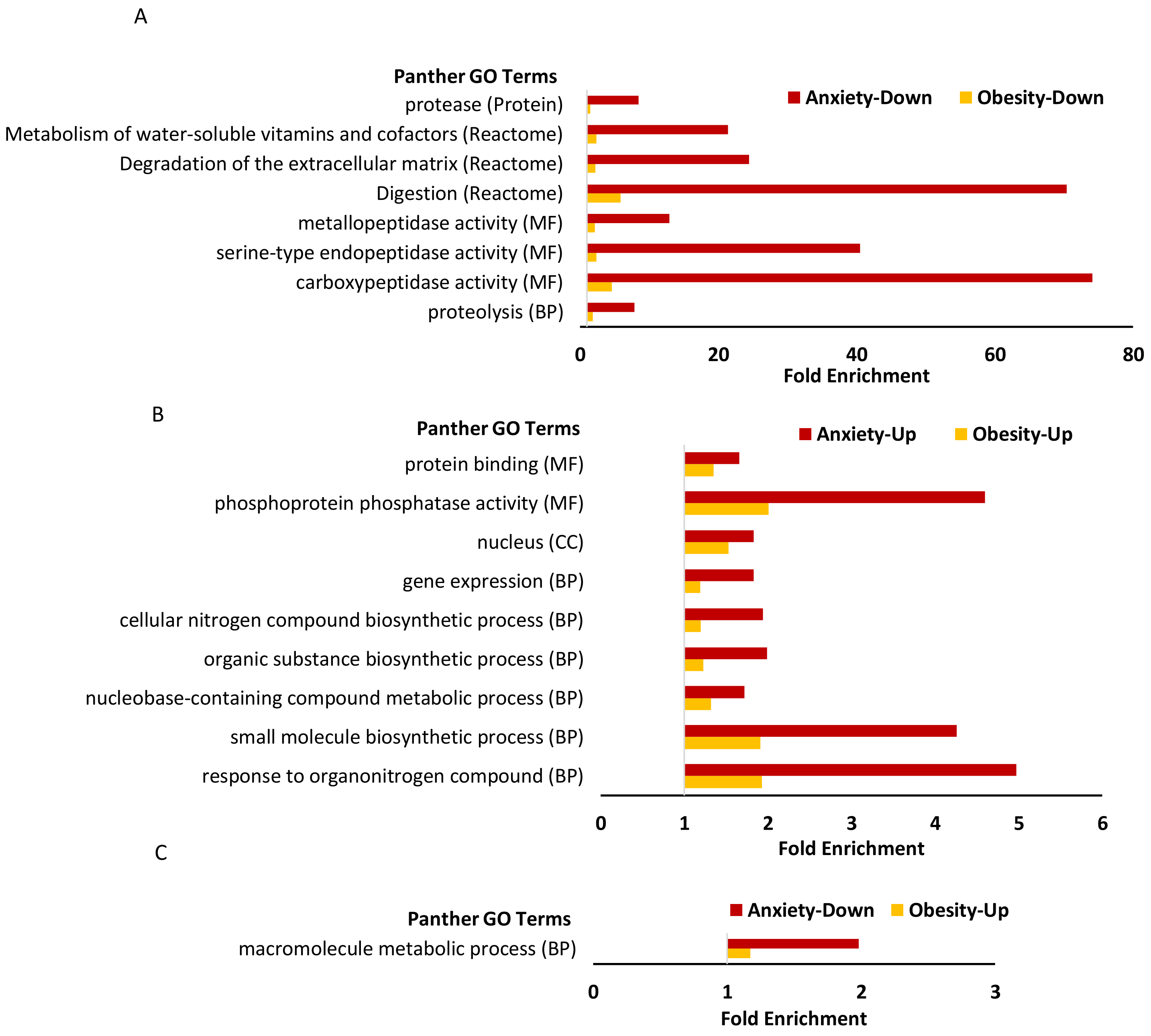

Figure S2. Panther Gene ontology (GO) terms that were overrepresented among anxiety and obesity DE genes - that were common to A) downregulated genes in both anxiety and obesity models B) upregulated genes in both anxiety and obesity models C) downregulated genes in anxiety and upregulated genes in obesity models. Reference genes were all genes in Danio rerio database. Fisher's Exact Test. Correction by FDR<0.05.

A)

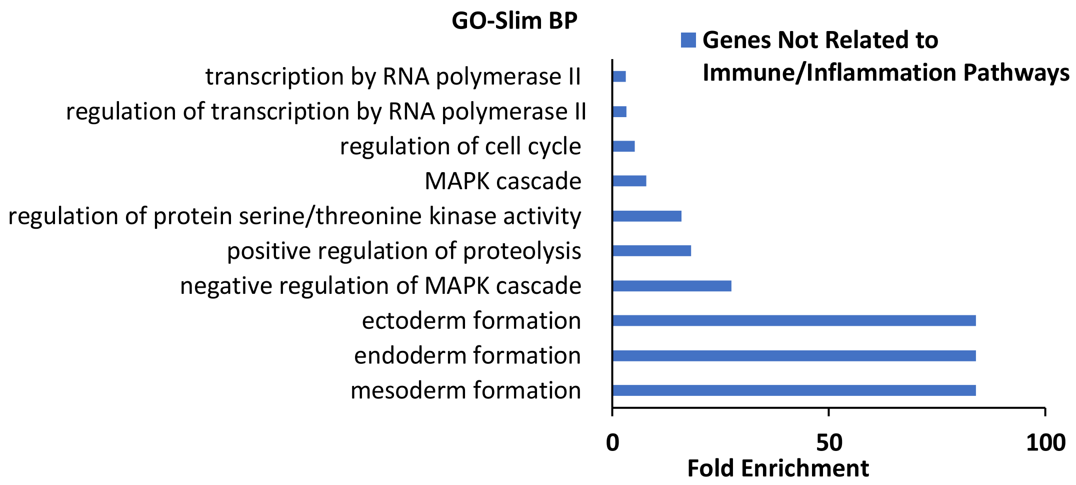

B)

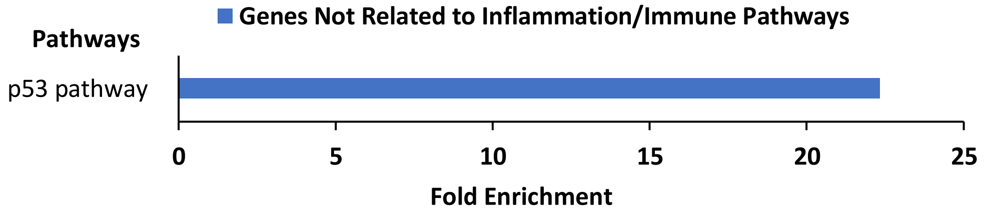

Figure S3. Panther Gene ontology (GO) terms overrepresented among non-immune genes of the anxiety-obesity inversely regulated genes. A) GO-Slim Biological Process (BP). B) Pathways. Reference genes were all genes in Danio rerio database. Fisher's Exact Test. Correction by FDR<0.05.

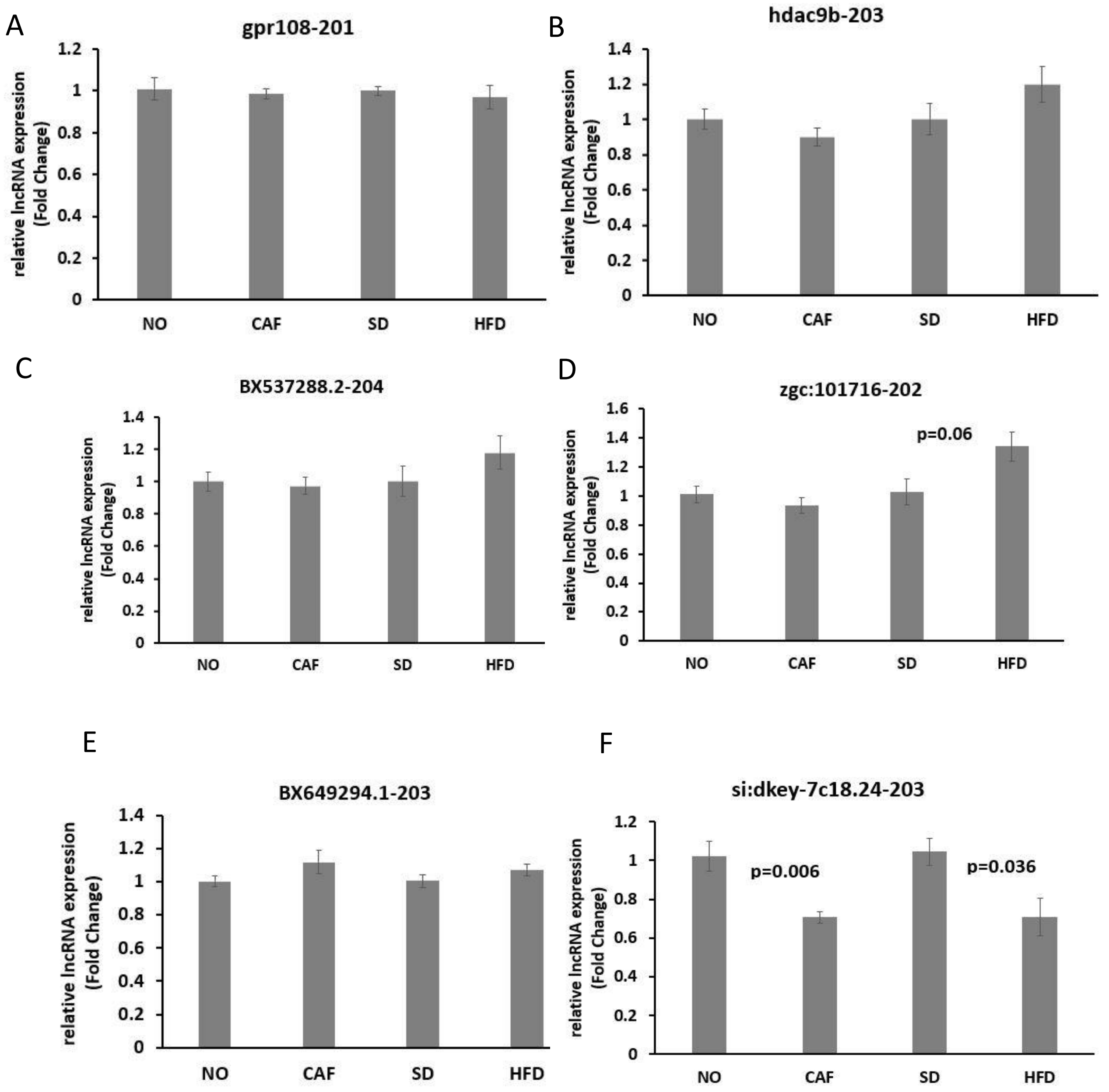

Figure S4. Both RNA-seq and qRT-PCR validated processed transcript lncRNA transcript, si:dkey-7c18.24-203. Graphs A and B are results of retained intron lncRNAs and graphs C-F are results of processed transcript RNAs. Mean ± SE, n= 5-8 replicates (10-30 larvae/replicate), 2-3 separate experiments, Student's t-Test. Reference genes were eef1a1l1 and actb2.

Table S3. Characteristics of lncRNAs

Characteristics of DE lncRNAs *						
**Gene name**	**Transcript name**	**Chromosome number**	**Strand**	**Transcript length (bps)**	**Number of exons**	**Gene length (kb)**
**CABZ01048956.1**	CABZ01048956.1-203	12	Reverse	4253	8	12.11
**CR926130.2**	CR926130.2-201	7	Forward	10347	11	28.5
**FO834828.2**	FO834828.2-201	1	Forward	4754	9	9.23
**CR926130.2**	CR926130.2-202	7	Forward	7308	7	17.61
**FO681323.1**	FO681323.1-202	1	Forward	4044	23	10.93
**FO904966.2**	FO904966.2-201	12	Forward	2588	2	17.5
**AL954191.1**	AL954191.1-201	5	Forward	4865	6	12.76
**si:dkey-7c18.24**	si:dkey-7c18.24-203	20	Forward	1557	3	1.78

* From https://www.ensembl.org/index.html, Zebrafish (GRCz11)

Table S4. LincRNA neighboring mRNAs

**lincRNA transcript name**	**mRNA neighbor(s)**	**mRNA name/function**	**Human symbol orthologue (DIOPT)**	**ZNC symbol**
**CABZ01048956.1-203**	BC154663	zymogen granule membrane glycoprotein 2		
	BC133062	zgc:153932	GP2	
**CR926130.2-201**	BC154840	unknown protein		epdl1
	FJ392620	uo:ion005 mRNA, partial sequence.		epdl1
	BC091686	cDNA clone IMAGE:7146140		
**FO834828.2-201**	BC124496	calmegin	CLGN	clgn
	BC071438	NMDA receptor-regulated gene 1b	NAA15	naa15b
	AY391464	transcriptional coactivator tubedown-100	NAA15	naa15b
	BC044392	NMDA receptor-regulated gene 1b	NAA15	naa15b
	BC049483	NMDA receptor-regulated gene 1b -WARNING-chimeric clone	NONE found	
	BC124711	zgc:153642		zgc:153642
	BC078402	zgc:91963	HTRA3	htra3a
**CR926130.2-202**	BC154840	unknown protein		epdl1
	FJ392620	uo:ion005 mRNA, partial sequence.		epdl1
	BC091686	cDNA clone IMAGE:7146140		
**FO681323.1-202**	BC071438	NMDA receptor-regulated gene 1b	NAA15	naa15b
	AY391464	transcriptional coactivator tubedown-100	NAA15	naa15b
	BC044392	NMDA receptor-regulated gene 1b	NAA15	naa15b
	BC049483	NMDA receptor-regulated gene 1b -WARNING-chimeric clone		
**lincRNA transcript name**	mRNA neighbor(s)	mRNA name/function	Human symbol orthologue (DIOPT)	ZNC symbol
**FO904966.2-201**	BC154663	zymogen granule membrane glycoprotein 2		
	BC133062	zgc:153932	GP2	

**AL954191.1-201**	BC159267	Purine-rich element binding protein B		LOC5648 40
	BC167481	si:dkey-202n14.1		purba
	BC162478	si:dkey-202n14.1,		
	BC162482	si:dkey-202n14.1		purba
	BC049019	H2A histone family, member V	H2AFV	h2az2a
	BC154791	H2A histone family, member V	H2AFV	h2az2a
	AF414110	histone variant H2A.F/Z mRNA	H2AFV	h2az2a
	BC056314	zgc:65851		zgc:65851
	BC065939	zgc:65851		zgc:65851

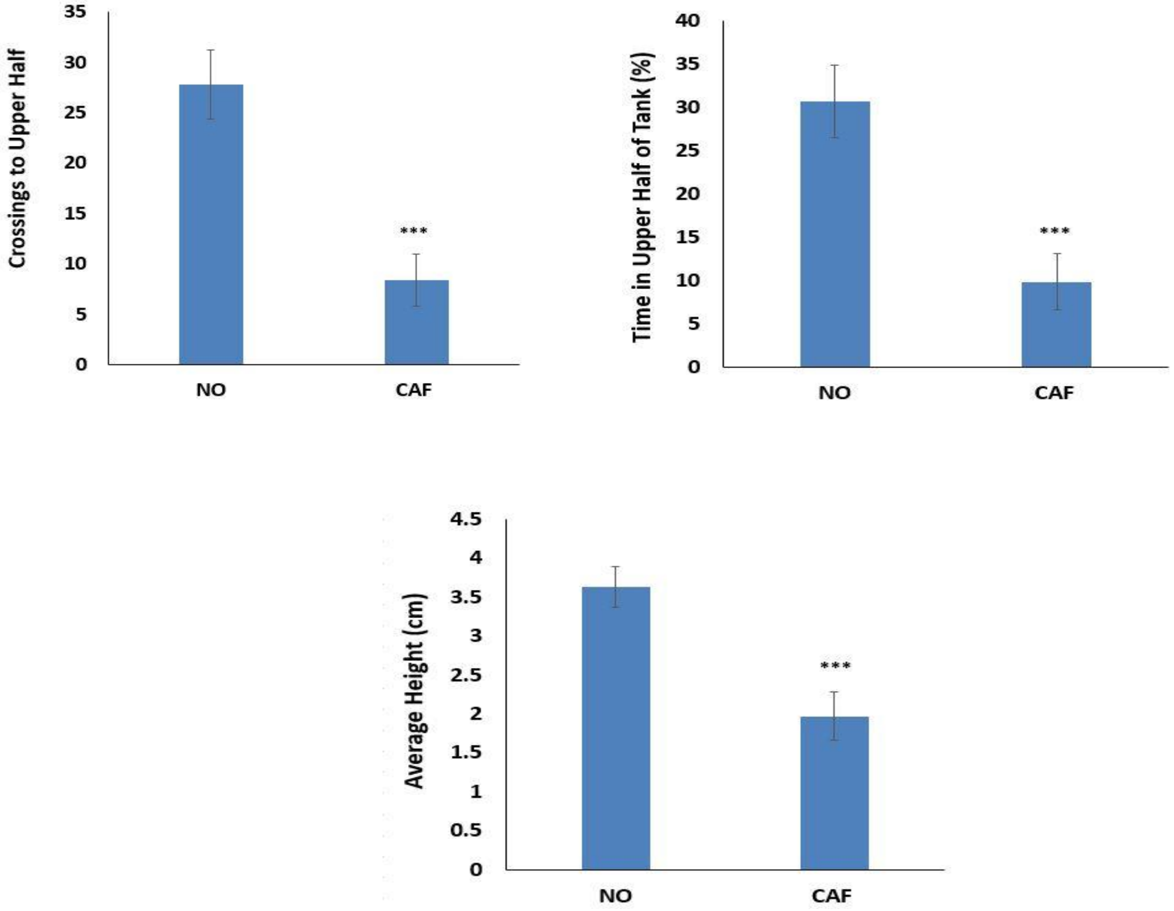

Figure S5. – Caffeine exposure induced strong anxiety-like behavior in adult zebrafish. NO – no treatment, CAF – caffeine exposure, NO F – non-treated females, CAF F – caffeine-exposed females, NO M – non-treated males and CAF M – caffeine-exposed males. Mean ± SE, n=12-14 males and female fish in 3 independent experiments (each experiment had both males and females). Student's t-Test, *** - $p<0.005$

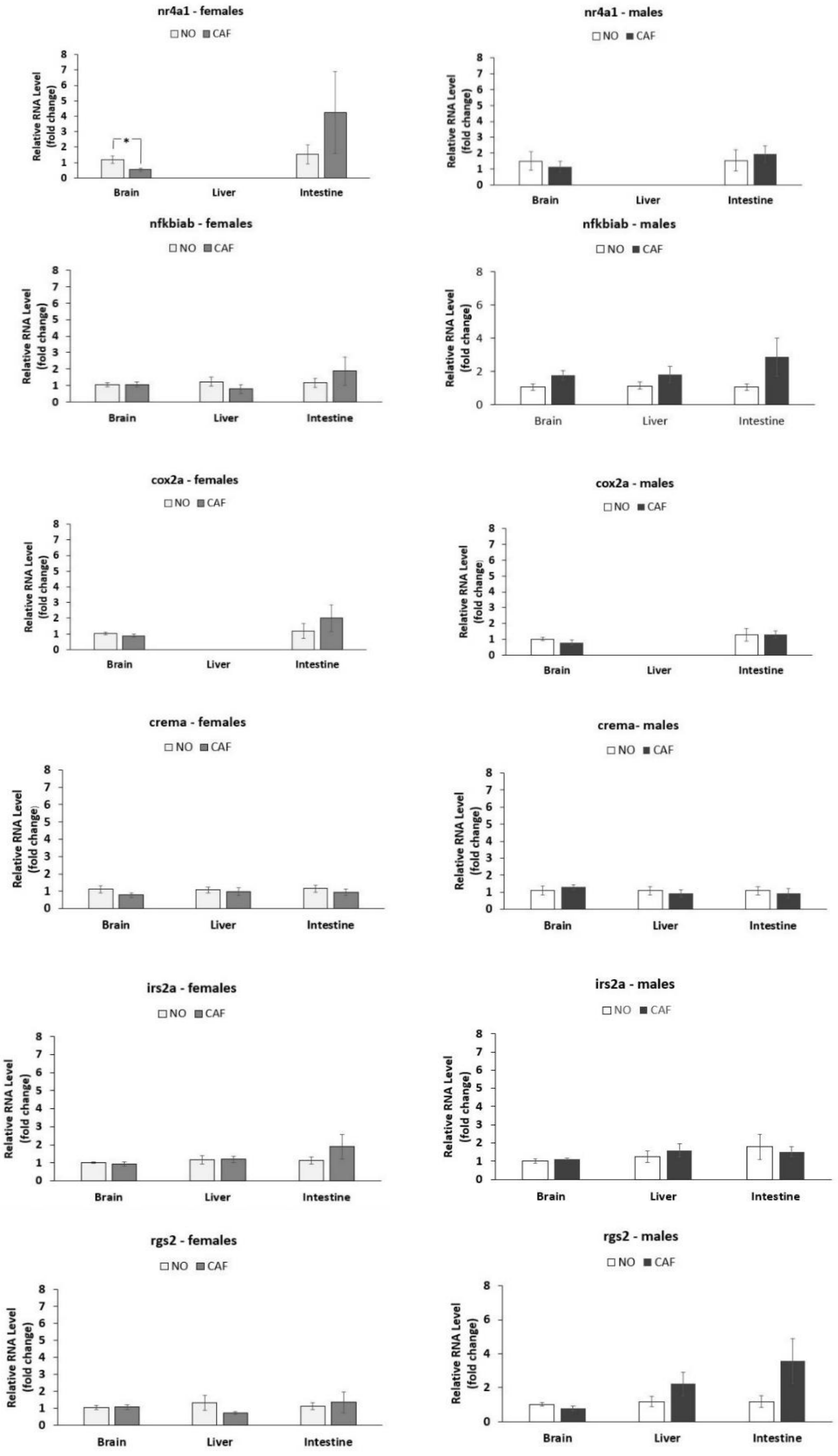
nr4a1 - females
NO CAF
Relative RNA Level (fold change)
*
Brain Liver Intestine
nr4a1 - males
NO CAF
Relative RNA Level (fold change)
Brain Liver Intestine
nfkbiab - females
NO CAF
Relative RNA Level (fold change)
Brain Liver Intestine
nfkbiab - males
NO CAF
Relative RNA Level (fold change)
Brain Liver Intestine
cox2a - females
NO CAF
Relative RNA Level (fold change)
Brain Liver Intestine
cox2a - males
NO CAF
Relative RNA Level (fold change)
Brain Liver Intestine
crema - females
NO CAF
Relative RNA Level (fold change)
Brain Liver Intestine
crema- males
NO CAF
Relative RNA Level (fold change)
Brain Liver Intestine
irs2a - females
NO CAF
Relative RNA Level (fold change)
Brain Liver Intestine
irs2a - males
NO CAF
Relative RNA Level (fold change)
Brain Liver Intestine
rgs2 - females
NO CAF
Relative RNA Level (fold change)
Brain Liver Intestine
rgs2 - males
NO CAF
Relative RNA Level (fold change)
Brain Liver Intestine

Figure S6. Transcripts upregulated in caffeine-treated larvae were not upregulated in organs of middle-aged caffeine-treated adult males and females. RNA Mean ± SE, n= 4-5 male fish and 7-9 females in each treatment, 3 independent experiments, with males and females in each. The reference genes were eef1a1l1 and actb2. Student's t-Test.

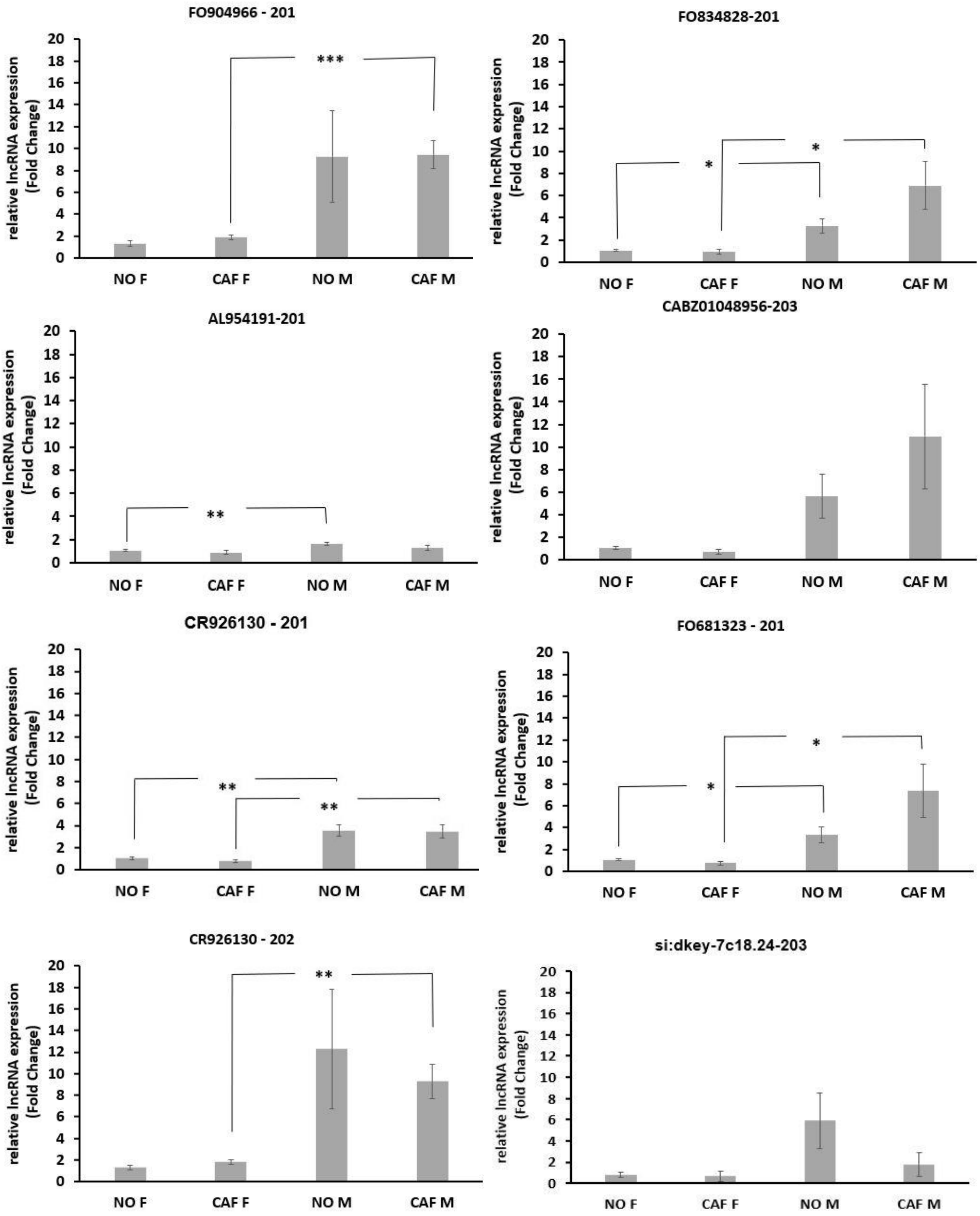

Figure S7. lncRNAs were more frequently expressed in males than in females. All graphs presented are lincRNAs except for that of si:dkey-7c18.24-203, which is a processed transcript lncRNA. NO F – no treatment females, CAF F – caffeine-exposed females, NO M – no treatment males and CAF M – caffeine-exposed males. Mean ± SE, n=5-7 individual fish, 2 independent experiments. The reference genes were eef1a1l1 and actb2. Student's t-Test, * - $p<0.05$, ** -$p<0.01$, *** - $p<0.005$

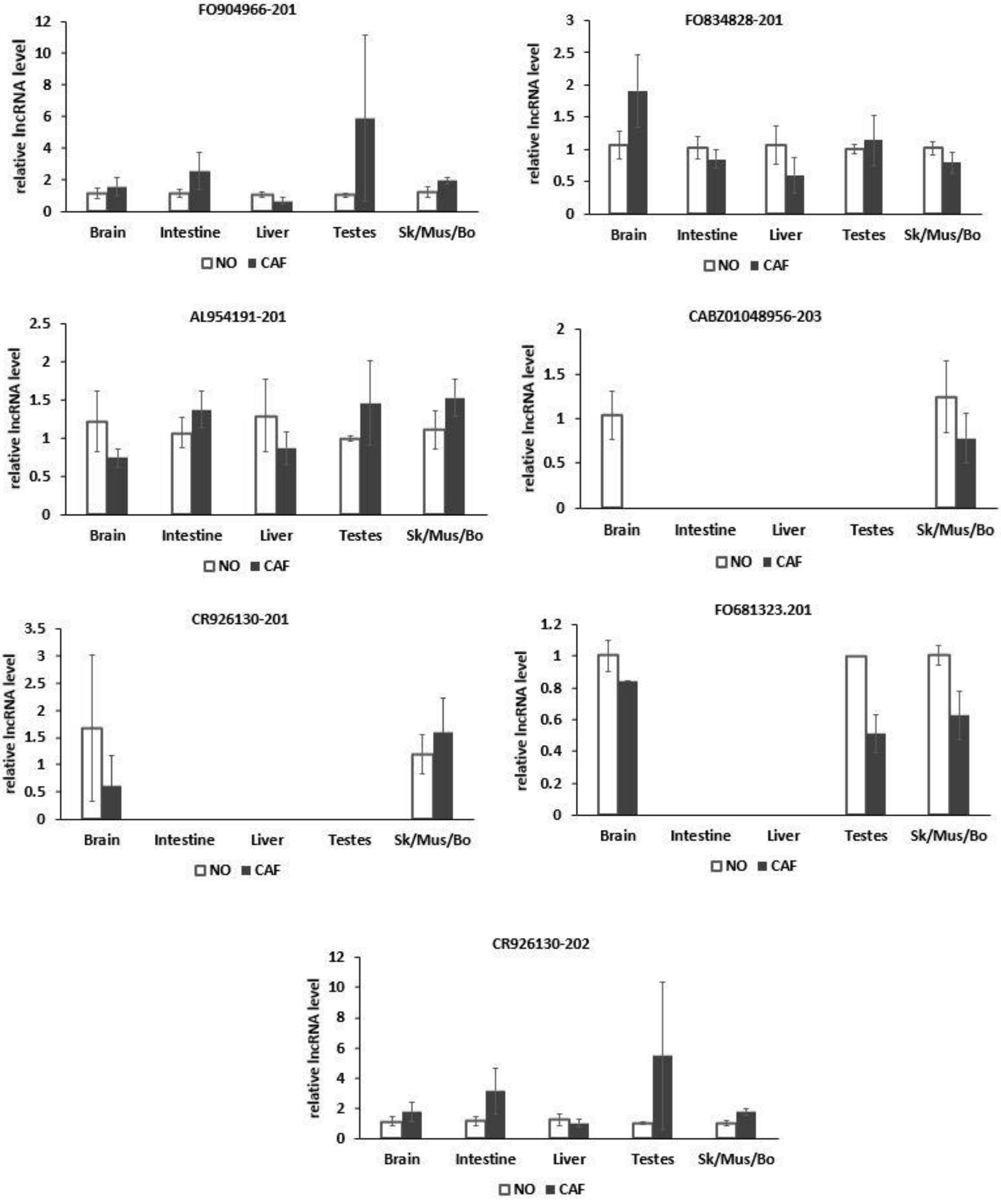

Figure S8. Certain lincRNAs were expressed in all tested organs of adult zebrafish. All graphs present lincRNAs. Two independent experiments. n=4-5 individual male fish. The reference genes were eef1a1l1 and actb2. Mean ± SE, Student's t-Test.
